# East Asian warm season temperature variations over the past two millennia

**DOI:** 10.1038/s41598-018-26038-8

**Published:** 2018-05-16

**Authors:** Huan Zhang, Johannes P. Werner, Elena García-Bustamante, Fidel González-Rouco, Sebastian Wagner, Eduardo Zorita, Klaus Fraedrich, Johann H. Jungclaus, Fredrik Charpentier Ljungqvist, Xiuhua Zhu, Elena Xoplaki, Fahu Chen, Jianping Duan, Quansheng Ge, Zhixin Hao, Martin Ivanov, Lea Schneider, Stefanie Talento, Jianglin Wang, Bao Yang, Jürg Luterbacher

**Affiliations:** 1 Department of Geography, Climatology, Climate Dynamics and Climate Change, Justus Liebig University Giessen, Giessen, Germany; 2 University of Bergen, Department of Earth Science and Bjerknes Centre for Climate Research, Allégt. 41, NO-5020 Bergen, Norway; 3 Department of Energy, Renewable Energy Section, Research Center for Energy, Environment and Technology (CIEMAT), Madrid, Spain; 4 Department of Physics of the Earth and Astrophysics, IGEO (UCM-CSIC), Universidad Complutense de Madrid, Madrid, Spain; 5 Institute of Coastal Research, Helmholtz-Zentrum Geesthacht, D-21502 Geesthacht, Germany; 6 Max-Planck Institute for Meteorology, Bundesstrasse 53, D-20146 Hamburg, Germany; 7 Department of History, Stockholm University, Stockholm, Sweden; 8 Bolin Centre for Climate Research, Stockholm University, Stockholm, Sweden; 9 Center for Earth System Research and Sustainability, CliSAP, University of Hamburg, Hamburg, Germany; 10 MOE Key Laboratory of Western China’s Environmental System, Lanzhou University, Lanzhou, 730000 China; 11 State Key Laboratory of Vegetation and Environmental Change, Institute of Botany, Chinese Academy of Sciences, 100093 Beijing, China; 12 Institute of Geographic Sciences and Natural Resources Research, Chinese Academy of Sciences, 11A, Datun Road, Chaoyang District, Beijing, 100101 China; 13 Key Laboratory of Desert and Desertification, Northwest Institute of Eco-Environment and Resources, Chinese Academy of Sciences, Lanzhou, China; 14 Centre for International Development and Environmental Research, Justus Liebig University Giessen, Giessen, Germany

## Abstract

East Asia has experienced strong warming since the 1960s accompanied by an increased frequency of heat waves and shrinking glaciers over the Tibetan Plateau and the Tien Shan. Here, we place the recent warmth in a long-term perspective by presenting a new spatially resolved warm-season (May-September) temperature reconstruction for the period 1–2000 CE using 59 multiproxy records from a wide range of East Asian regions. Our Bayesian Hierarchical Model (BHM) based reconstructions generally agree with earlier shorter regional temperature reconstructions but are more stable due to additional temperature sensitive proxies. We find a rather warm period during the first two centuries CE, followed by a multi-century long cooling period and again a warm interval covering the 900–1200 CE period (Medieval Climate Anomaly, MCA). The interval from 1450 to 1850 CE (Little Ice Age, LIA) was characterized by cooler conditions and the last 150 years are characterized by a continuous warming until recent times. Our results also suggest that the 1990s were likely the warmest decade in at least 1200 years. The comparison between an ensemble of climate model simulations and our summer reconstructions since 850 CE shows good agreement and an important role of internal variability and external forcing on multi-decadal time-scales.

## Introduction

Over the past two decades, many proxy-based temperature reconstructions at hemispheric and continental scale covering the past one to two millennia have been published^1–4^. Northern Hemisphere (NH) temperature reconstructions generally show a warm period ca. 1–300 CE, followed by a cooler period ca. 300–700 CE, warm conditions from ca. 800 to 1250 CE, and a cooler climate from about 1300 CE until the end of the 19th century^1,2^. The reconstructions agree with instrumental observations in showing a rapid temperature increase in the late 20th century with exceptionally high temperatures in the last few decades in the context of the past centuries^1,2,5,6^. Regional temperature reconstructions from East Asia/China show a similar behavior to that of the NH, but they disagree on whether the warming over the last few decades has been unprecedented or not in the context of the past^7,8^.

**Figure 1:**
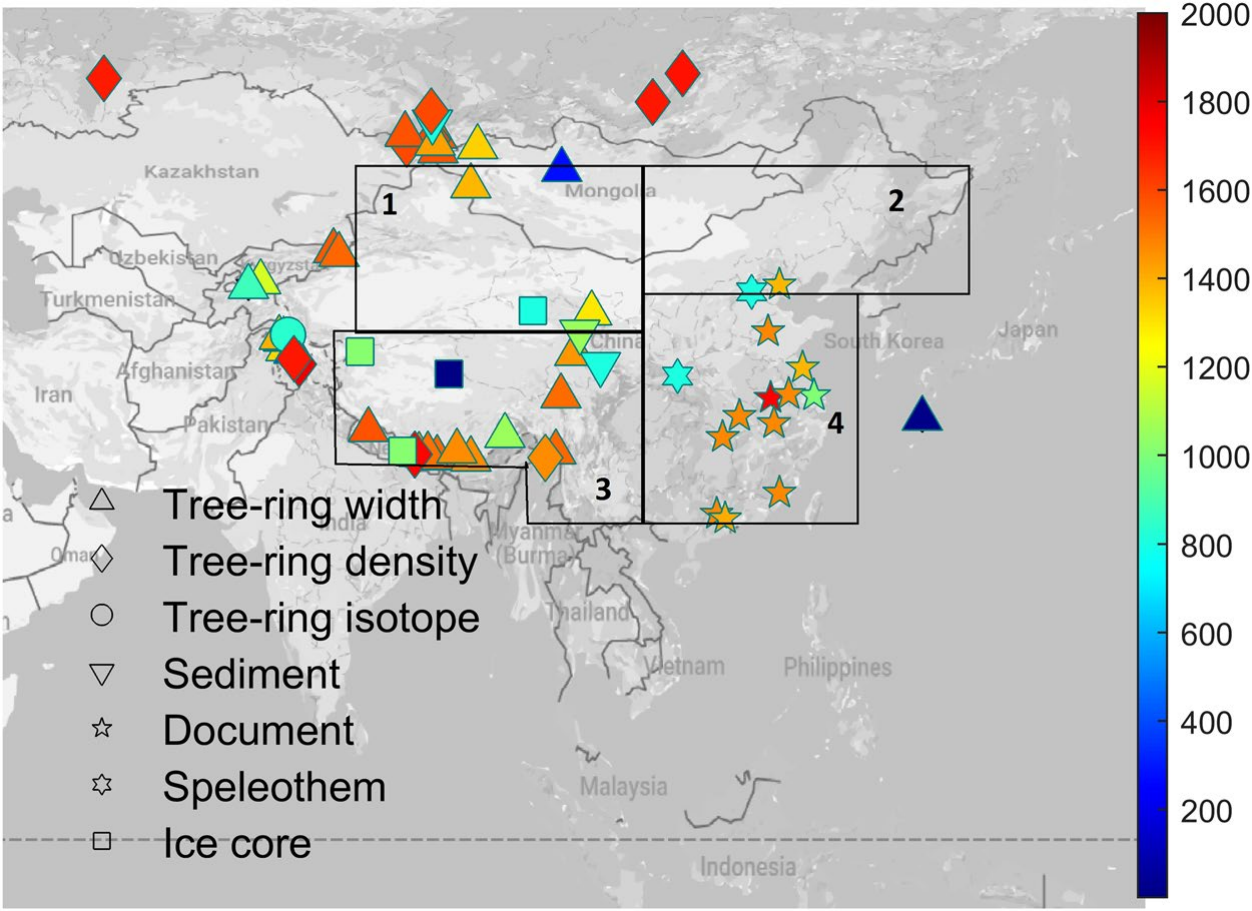
Locations of the proxies used in this study; the color indicates the starting year of the proxy record. The four selected sub-regions (with different climate) mainly include Northwest China-western Mongolia (marked as 1), Northeast China-eastern Mongolia (2), Tibetan Plateau (3) and Southeast China (4). The figure was generated using Matlab 2015b (http://www.mathworks.com/). The map in the figure was queried from Google Static Map APIs (http://code.google.com/apis/maps/).

Climate field reconstructions (CFRs), complementing regional average reconstructions, offer temporal and also spatially resolved information over past temperature variability. So far, two field temperature reconstructions over East Asia have recently been published^9,10^. Cook *et al*.^9^ reconstructed summer temperatures at annual resolution back to 800 CE using 229 tree-ring chronologies and applying a point-by-point regression method. Shi *et al*.^10,11^ generated an annual-resolved temperature field reconstruction covering the last millennium based on 418 proxy records (392 being tree-ring chronologies), applying the Regularized Expectation Maximization method with Truncated Total Least Squares (RegEM-TTLS). The CFR techniques used in both of the two reconstructions are based on multivariate linear regression approaches, which are based on calibrating proxy records against instrumental data^12–17^. Apart from linear regression approaches, the Bayesian hierarchical model^18^ (BHM), a probabilistic approach, is another CFR method that has been used to reconstruct the past regional temperature variability for Europe and the Arctic^6,19,20^. The advantages of the Bayesian method include: a more comprehensive assessment of uncertainties, no need for grid infilling (for instrumental and/or proxies), transparent assumptions about the spatial-temporal properties and no dependence on long-distance correlations that might not be stable in time. In addition, probabilistic ensembles of equally likely reconstructions provide unique means to evaluate e.g. trends or extreme periods^20^. BHM is suitable for estimating uncertainties of reconstructed temperatures^21,22^. In this study, we applied BHM to 59 selected temperature-sensitive proxy records (see Fig. 1 and the methods section) over East Asia (60°–160°E/10°–60°N) and generated a new warm-season (May-September) temperature reconstruction for the past 2000 years with decadal resolution and associated uncertainties. We compare our findings with earlier temperature reconstructions and with an ensemble of state-of-the-art climate model simulations of temperature over the period 850–2000 CE in order to assess the influence of changes in external forcing and the simulated internal climate variability over East Asia.

## Results and Discussion

**Figure 2:**
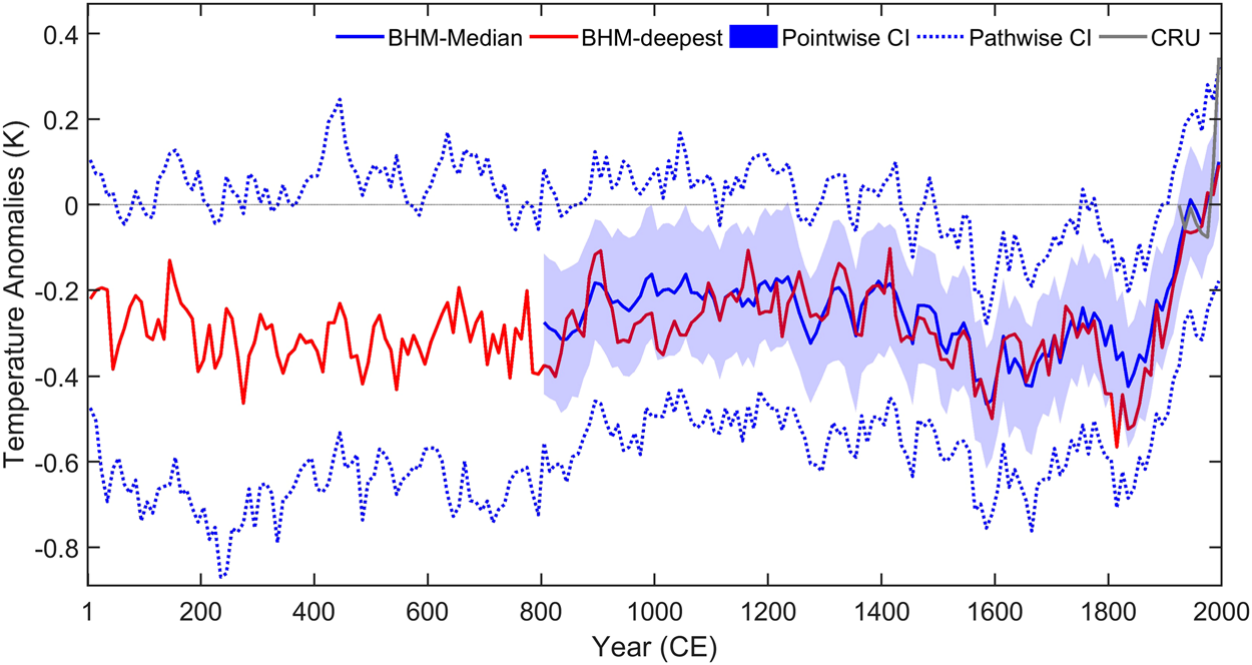
Reconstructed area-weighted decadal temperature anomalies (w.r.t. 1961–1990 CE) using the full proxy network and Bayesian hierarchical model with instrumental data excluded. Solid blue line: the median values; Solid red line: the deepest curve; blue shades: 90% point-wise confidence intervals; dashed blue line: 90% path-wise confidence intervals; solid gray line: instrumental warm season temperature (Jones *et al*.^39^).

Two regional average temperature time-series (anomalies w.r.t. 1961–1990 CE) together with their uncertainties are presented in Fig. 2: Point-wise median values covering the period 801–2000 CE, denoted as BHM-median; and path-wise median values covering the period 1–2000 CE, denoted as BHM-deepest. The regional average temperature reconstruction over the period 801–2000 CE is tested as robust against changes in the proxy-data network and methodology (the Pearson correlation coefficients are higher than 0.86 between the BHM and the composite reconstructions; see the Methods section for more details). BHM-median represents the best temperature estimates for each decade, but may suffer from loss of variance for early centuries and over some specific areas due to sparse proxy coverage. However, this potential issue has been solved for BHM-deepest. BHM-deepest is the median trajectory of reconstructed temperature variations if considering each ensemble member as a possible trajectory of past temperature (illustrated in the methods subsection “Deepest curve and path-wise confidence intervals”). Compared with BHM-median, BHM-deepest exhibits a higher temperature variability. These two BHM reconstructions (BHMs) share a large part of the inter-decadal temperature variability (*r* = 0.83, *df* = 118, *p* < 0.01) over their common period 801–2000 CE. The BHM reconstructions indicate that relatively warm East Asia warm-season conditions prevailed from the beginning of the Common Era until the 3^rd^ century CE, and were followed by generally cooler conditions in the subsequent centuries (Fig. 2). Rather high temperatures are found ca. 900–1200 CE, in the early 14^th^ century and at the turn of the 15^th^ century. The relatively warm conditions were followed by overall cooler conditions from 1450–1850 CE. Since the 1850s, the warm-season temperature has been increasing, a trend that holds until the present with a warming pause between ca. 1940 and 1970 CE. This is consistent with instrumental observations. Joint information from our regional average temperature reconstruction and the instrumental data indicates that the 1990s were likely the warmest decade in the last 2000 years, as it exceeds the upper boundary of 90% path-wise confidence intervals (Fig. 2).

**Figure 3:**
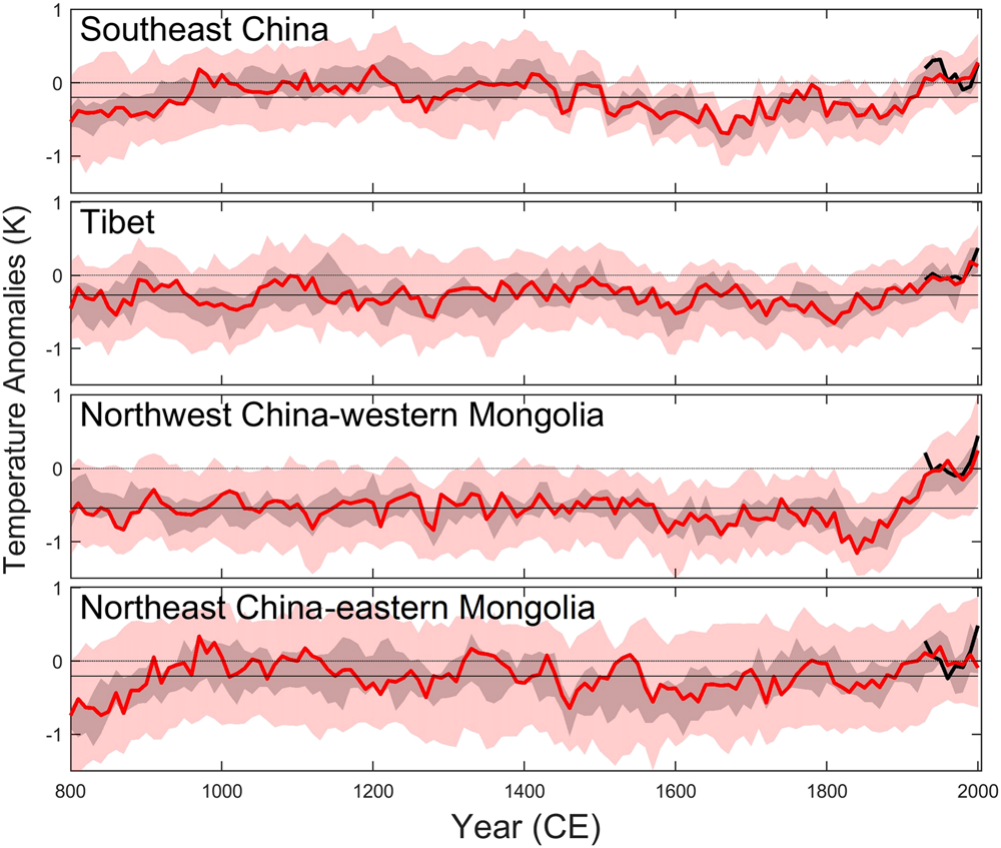
Reconstructed area-weighted temperature anomalies (with respect to 1961–1990 CE) using the 59 proxy record network and Bayesian hierarchical model with instrumental data excluded in the selected regions. Red curve: the deepest curve; thick black curve: decadal mean instrumental temperature; light black line: regional average temperature with respect to the whole period (801–2000 CE); gray shades: the variance range of the ten deepest curves; red shades: 90% path-wise confidence intervals.

The BHM spatial temperature reconstructions over four sub-regions indicate that the warmer conditions were spatially heterogeneous from the 9^th^ century to the end of the 13^th^ century (Fig. 3). For instance, warmer conditions can be found over south-east China, but anomalous lower temperatures were reconstructed over the Tibetan Plateau (from mid-10^th^ century to end of the 11^th^ century and again from mid of the 12^th^ century to end of the 13^th^ century). In contrast, the cold conditions from the 16^th^ century to the 19^th^ century covered almost the whole of East Asia. Particularly cold periods are prominent in the mid-17^th^ century over south-east China and in the 19^th^ century over northwest China and western Mongolia. The reconstructions indicate that, the temperature in the 1990s is likely to be the highest in the last millennium over the Tibetan Plateau^23,24^, northwest China and western Mongolia^25^. The warm conditions in the 1990s are not exceptional over south-east China. Equally warm conditions prevailed during medieval times^26^ and during the period 1921–1940 CE.

### Comparison with China/ East Asia temperature reconstructions

**Figure 4:**
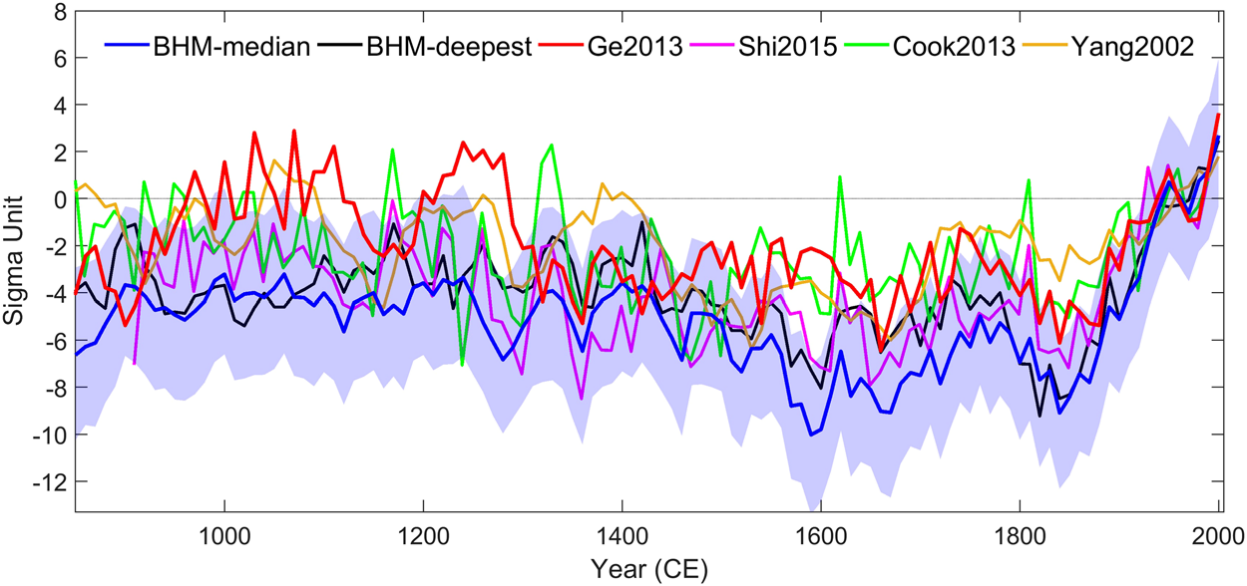
BHM reconstructions from this study in comparison with published evidence for East Asia (all reconstructions are presented at decadal resolution and standardized with respect to 1921–1990 CE). Dashed black lines are 90% point-wise confidence intervals of BHMs.

We compared the BHMs reconstructions with four other regional average temperature reconstructions over China / East Asia (Yang^27^, Cook^9^, Ge^26^, and Shi^10^, see Table 1 for a description of reconstructions and cross-correlation analysis) for the last 1150 years. The reconstructions differ regarding their amplitudes of variation for the instrumental period (Fig. S6). For a fair comparison, all reconstructions were standardized with respect to the instrumental period 1921–1990 CE (Fig. 4).

**Table 1:** (a) Selected published temperature reconstructions over China or Asia.

(a)						
Reconstruction name	Number of proxy data	Method	Season	Region	Climate field reconstruction?	Publication
Shi2015	418 multi-proxy (including 392 tree-ring chronologies)	Regularized expectation maximization algorithm	Summer	Asia	Yes	^10^
Cook2013	229 tree-ring proxies	point-by-point regression	Summer	East Asia	Yes	^5,9^
Ge2013	22 multi-proxy	Composite method	Annual	China	No	^26^
Yang2002-weighted	9 multi-proxy	Composite method	Annual	China	No	^27^
**(b)**						
**Cor**.	**BHM-median**	**BHM-deepest**	**Shi2015**	**Cook2013**	**Ge2013**	
BHM61-deepest	**0.83**					
Shi2015	**0.68**	**0.52**				
Cook2013	**0.46**	**0.40**	**0.79**	—		
Ge2013	**0.55**	**0.44**	**0.47**	0.24	—	
Yang2002	**0.62**	**0.45**	**0.45**	**0.28**	**0.51**	

(b) Pearson correlation analysis among different reconstructions of decadal temperature for the period 901-1990 CE, df = 107 (p<0.01 are in bold). The critical value for significant correlation at the level p = 0.01 is 0.25. It should be kept in mind that the real p-value is probably much higher than 0.01 due to strong autocorrelation in the reconstructions.

The reconstructions roughly agree on the timing of three characteristic periods: a multi-centennial warm period ca. 900–1200 CE, the cold “Little Ice Age” ca. 1450–1850s CE and a continuous warming after the 1850s. They differ in terms of the shape and strength of temperature variations and the timing of cold and warm periods. Moreover, Shi2015 and our new BHM reconstruction suggests, that China/East Asia experienced likely the warmest decade during the 1990s for more than 1100 years.

Ge2013 and our BHMs share a large portion of proxies and show a similar temperature variability for the last half millennium (with the exception that the BHMs reveal a stronger centennial scale variability). They differ in both the shape and strength of the low-frequency variability before the 13th century. The BHMs suggest a modest warming and relatively weak temperature fluctuation during the medieval time while Ge2013, Cook2013 and Yang2002 suggest that a few decades during the MCA were as warm as the 1990s. Generally, the proxy data networks become sparser back in time and the reconstructed variance is increasingly sensitive to the influence of individual proxy records. The strong warming and temperature fluctuation of Ge2013 before the 15th century seem to be mainly influenced by two low-resolution proxies (>10a, “Hong2000” and “Ge2003”) over eastern China^26,28^. It is difficult to estimate the biases in the two proxies but they might be over-weighted when we consider the spatial decorrelation length of temperature. The better spatial coverage in the proxy network applied for the BHMs may provide a more robust temperature estimation in this early period compared to Ge2013.

The discrepancies between the reconstructions may be from different (but partly overlapping) proxy networks, different data selection strategies, and different reconstruction methods. But most important is the data selection step. And from this perspective, Ge2015 and our BHMs are probably more reliable than the others. Looking into the sub-regional temperatures reconstructed in Ge *et al*.^26,28^, we find agreement with our study: western China experienced warmer conditions in the 1990s compared to any decade of the MCA. Further, eastern China may have experienced similar warm conditions climate during some decades of the MCA.

Shi2015 and Cook2013 are based on climate field reconstructions. Cook *et al*.^9^ used only tree-ring chronologies mainly from high latitudes or western mountainous areas. Shi *et al*.^10^ employed tree-ring-dominant (>93%) proxy records (all 341 tree-ring chronologies provided by Cook *et al*.^9^ and another 51 tree-ring chronologies obtained through private communication). As tree-ring growth is influenced by several environmental factors during the growing season, it is necessary to check their temperature sensitivity. Note that, most of the tree ring chronologies were also used to reconstruct the PDSI (Palmer Drought Severity Index) over monsoon Asia^29^. Cook2013 was pointed out as having “substantial uncertainties in low frequencies” by the PAGES 2K Consortium^5^. This shortcoming is addressed by removing tree-ring chronologies with low temperature sensitivity and by adding data from other proxy archives. Yang2002 and Ge2013 are regional average temperature reconstructions based on the composite methods using both a smaller number of proxies.

### Comparison with GCM simulations

**Figure 5:**
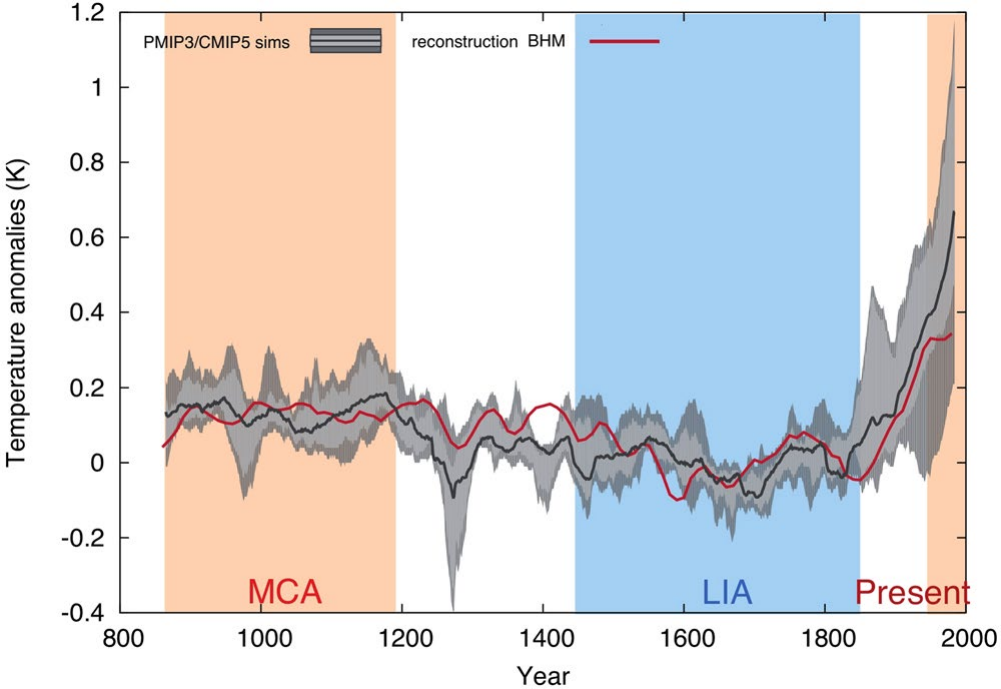
Simulated and reconstructed East Asia warm-season land temperature anomalies (with respect to 1500–1850 CE) for the last 1200 yr (850–1980; The decadal-resolute reconstructions are 3 decades moving average, and the annual model data are 31-yr filtered). BHM59 reconstructed temperature are shown dark green while BHM13 appear in light green over the spread of model run. The ensemble mean (heavy black line) and the band accounting for 50% and 80% (shading) of the spread are shown for the model ensemble.

Figure 5 shows the BHM reconstructions and the median of the ensemble of simulations along the 10, 25, 75 and 90 percentile uncertainties based on the ensemble spread^30^. This spread reflects differences in the models and the individual forcing implementation and also internal variability in decadal to multi-decadal temperature variations. The latter can be seen in multiple realizations of the same model and it has been shown that individual ensemble members can deviate substantially from the ensemble mean even under strong external forcing^31^. Reconstructions and simulations are filtered using a 31-yr moving average filter to highlight the low-frequency variability and to compare temperature variations influenced by external forcing.

The reconstruction for the East Asian temperatures correlates well with the median of the ensemble of simulations (*r* = 0.77, *df* = 113, *p* < 0.01). The external forcing used in the model experiment explains a significant fraction of East Asia warm season temperature variations during 850–2000 CE. The imprint of large volcanic eruptions (e.g. Samalas 1257 CE, Kuwae 1453 CE) is clearly visible, where simulations tend to show stronger short-term responses than reconstructions and instrumental observations^32^. Changes in solar activity can directly influence earth climate via thermal processes, changes in tropical convective activity or indirectly by changes in ozone photochemistry and related changes in stratospheric circulation eventually propagating into the troposphere^33,34^. Due to the comparatively small changes in solar activity during the last millennium the impact on climate might be seen as being supportive in amplifying prolonged cool phases in conjunction with increased volcanic activity. Solar forcing^33,34^ likely contributed to episodes of cooling during periods of low solar irradiance (Fig. S8): the Oort Minimum (ca. 1040–1080 CE), the Wolf Minimum (ca. 1280–1350 CE), the Spörer Minimum (ca. 1460–1550 CE), the Maunder Minimum (ca. 1645–1715 CE) and the Dalton Minimum (ca. 1790–1820 CE). However, as some solar minima coincide with strong volcanic events, it is difficult to distinguish their influence on multi-decadal temperature variability from the effects of solar forcing.

The simulations and the reconstruction agree on the timing and duration of the Medieval Climate Anomaly (MCA, ca. 900–1200 CE) and the Little Ice Age (LIA, ca. 1450–1850 CE) as well as the amplitude of the low-frequency variability. A few cooling intervals, possibly being due to low-solar-activity, in the simulations cannot be found in our East Asia regional average reconstruction. For instance, the simulated temperature minimum over the late Maunder Minimum (late 17^th^ century/early 18^th^ century) is not recorded in proxy records over East Asia; the simulated cold period during the MCA, attributed to the Oort Minimum, appeared over the Tibetan Plateau but not in the other regions of East Asia (see Fig. 3). Assuming the BHM reconstruction is the best currently available reconstruction, it suggests that the influence of solar forcing on regional decadal temperature variation may be much more heterogeneous than ensemble means of the GCMs. The more homogeneous temperature patterns in response to forcing in the model simulations have also been demonstrated in the study from PAGES 2k–PMIP3 group^35^.

The simulations and the reconstruction suggest a similar warming rate over the period 1850–1950 CE (0.04 °C /decade) and the temperature over the last thirty years of the 20^th^ century is likely the warmest 30-years in the last 1150 years. However, a few differences are noticeable: Firstly, the reconstruction suggests that the rapid warming started around mid of 19^th^ century^36^, that is two to three decades later than in the simulations (in which the warming started in the 1820s, the timing for the peak of Dalton minimum and Tambora eruption^33,34,37^). Secondly, there was no obvious multi-decadal time warming pause at the end of 19^th^ century in the reconstruction. Thirdly, the simulations do not show a warming pause during 1940–1970 CE, which can be seen in both the reconstruction and the instrumental temperature (Fig. 2). The simulated rapid warming is mainly attributed to the greenhouse gases (see Model settings in Table S2). However, anthropogenic aerosols might have also played an important role in the temperature variability in the second half of the 20th century^38^. We note that, for most of the pre-industrial last millennium (850–1850 CE), the BHM reconstruction lies within the multi-model ensemble spread (Fig. 5). Two exceptions are seen: The cooling at the end of the 16^th^ century is slightly larger in the reconstruction (possibly due to a cluster of volcanic eruptions). The reconstruction also suggests a warmer interval at the turn of the 15^th^ century while the simulations show a cooler climate driven by low-solar forcing.

**Figure 6:**
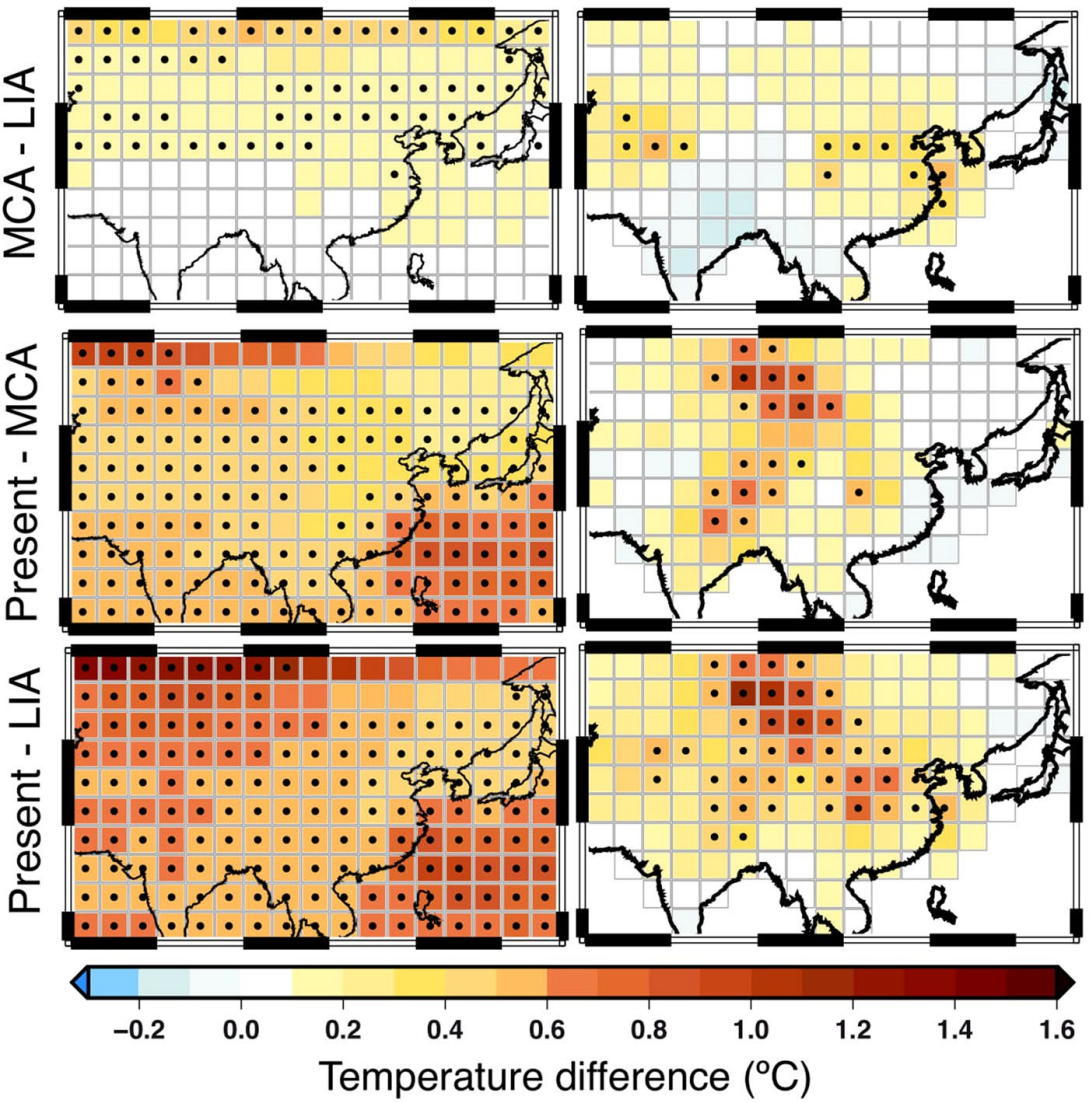
Simulated (left) and reconstructed (right) warm season (May–September) temperature differences for three periods: MCA (900–1200) minus LIA (1450–1850); present-day (1950–2000) minus MCA; and present-day minus LIA. Model temperature differences indicate average temperature changes in the ensemble of available model simulations. Reconstructed temperature differences with the BHM methods with full proxy network are shown in the right column. Simulations have been weighted by the number of experiments considered from each model (see Table S2 in the supplementary material). Dots indicate significant (p < 0.1) changes in the reconstructions; in the simulation ensemble a dot indicates at least 80% of agreement in depicting significant (p < 0.05) changes of the same sign. Figure was plotted using Generic Mapping Tools (http://gmt.soest.hawaii.edu/).

Similar to^6^, we compared the simulations with the reconstruction on the spatial temperature differences for the transitions: MCA (900–1200 CE) minus the LIA (1450–1850 CE), present-day (1950–2000 CE) minus the MCA and present-day minus the LIA (Fig. 6). The simulated differences are statistically significant at the 5% level at most of the grid-points, indicating a high agreement among the model ensemble members and suggesting that changes in external forcing have had a prominent influence on past East Asia warm-season temperature variations. Both the simulation ensemble mean and the reconstruction suggest that southeast China and the region north of 30 ° N experienced warmer climate during the MCA than during the LIA (top panels in Fig. 6). Moreover, the reconstruction and the simulation agree on the warmer conditions during the present time compared to the LIA (mid panels in Fig. 6). Disagreements between simulations and reconstructions can be found for the temperature difference between present-day and MCA (bottom panels in Fig. 6). The simulations show warmer conditions over the whole domain, similar to the temperature differences between the present-time and the LIA but with smaller anomalies. Our reconstruction indicates that the warming may be more spatially heterogeneous, i.e. warmer conditions over the middle part of the domain and cooler conditions over the lower reaches of the Yangtze River. Regional-scale differences are often observed comparing proxy-based climate reconstructions and model simulations^35^. The reconstructions are based on “real world” proxies, which allow an assessment of the true climate trajectory (taking into account the associated uncertainties in the reconstruction). The regional temperature series derived from climate simulations represent single trajectories that are consistent with changes in external forcings (orbital, volcanic, solar, greenhouse gases and land use) applied to the models as boundary forcing. The presence of internal climate variability, uncertainties in the external forcings as well as model errors limit temporal consistency between individual model trajectories and empirical reconstructions. For continental scales, the impact of changes in external forcings should be large enough to allow for a first order comparison. Still some characteristics (monsoonal changes in tropics, changes in extratropical circulation, changes in sea surface temperatures in adjacent continental areas etc.) can modify the direct impact of external forcings on East Asian temperatures in both, empirical reconstructions and model simulations.

We present a novel climate field reconstruction for East Asia using selected multi-proxy evidence. We extend available reconstructions to 2000 years and also assess uncertainties by applying a stochastic model for a multi-proxy network over a larger area. It generally compares well with the ensemble model simulation for the last 1150 years, which not only assigns the climate models confidence but also offers physical explanation for the past temperature evolution in the reconstruction. This gridded product will be used to analyze spatial modes of long-term temperature variability within the East Asia area and further compare with the simulations under various individual forcing, with the aim to assess for example the sub-regional effects of volcanic eruption, solar- and land-use change etc.

## Data and Methods

### Instrumental data

The instrumental temperature field used for the calibration of the Bayesian hierarchical model was derived from the global CRUTEM4v dataset, hereafter referred to as CRU^39^. This dataset has been used to calibrate proxy data in previous works (e.g. a European summer temperature reconstruction^6^).

This dataset comprises monthly mean temperature anomalies (with respect to 1961–1990 CE) with 5.0 ° × 5.0 ° resolution spanning the period 1850–2010 CE, but only data from the year 1921 onward have been used in this study as most meteorological stations are established after 1921 CE over East Asia. The region (60 ° − 160 ° E/10 ° − 60 ° N) comprises in total 132 grid points, excluding grid cells over the ocean or islands. As in^6^, gaps with missing observations were filled using the Regularized Expectation Maximization algorithm with ridge regression^40^ (RegEM). Then the warm season (May-September) mean temperature at each grid point and each decade was calculated using the infilled dataset for the comparison with the reconstructions. Non-infilled CRU was used as input data for the BHM reconstructions. Regional anomalies (w.r.t. 1961–1990 CE) of warm season temperature over East Asia using non-infilled CRU and infilled CRU are represented in Fig. S1 including the decadal-mean time series derived from the infilled CRU data.

Meteorological station data were used to select temperature-sensitive tree-ring proxy records. The station dataset includes monthly temperature data from 710 stations covering the period 1961–2000 CE, and the data were collected and processed by the National Meteorological Information Center of the China Meteorological Administration.

### Proxy data

Proxy data with at least decadal resolution were used, including tree-ring widths (TRW), tree-ring maximum latewood density (MXD), tree-ring isotopes, historical documentary data, ice cores, speleothems, lake sediments, and marine data. TRW and MXD proxy data were mainly taken from PAGES2k^5,9^ and a few MXD records were from the International Tree Ring Data Bank (ITRDB, http://www.ncdc.noaa.gov/paleo/treering.html).

TRW growth, and thus the TRW, is influenced by several environmental factors during the growing season, such as temperature and/or precipitation in water- and/or energy limited regions^41^. In contrast, MXD is generated at the end of the growing season and thus should not reflect a precipitation signal^42^. However, they still need to be checked for temperature sensitivity. To make sure the MXD and TRW data used for the reconstruction are temperature sensitive, the following steps were carried out:

1. The tree-ring records located over China or at the Chinese border were compared with the closest meteorological station data within 500 km at an annual scale covering the period 1961–1990 CE (This period is chosen while most meteorological stations over Tibetan Plateau are established after the late 1950s, and lots of TR proxy records do not cover the 1990s). Meteorological station data rather than CRU were used for the proxy-data screening because we found the tree-ring proxy records have a higher cross-correlation with near-by station data. A proxy record is retained when the Pearson correlation coefficient with the station data is higher than 0.25 (the critical value at one-tail 10% significance level for 30 years).
2. The tree-ring records without a nearby meteorological station were compared with CRU gridded data for the periods 1921–2000 CE and 1961–1990 CE. The proxy record is retained when one of the correlation coefficients is significant (higher than the critical value at a one-tail 10% significance level).
3. In data-rich regions, i.e. regions where more than one proxy record is available within 50 km, only a single record is kept. We chose the record that has higher correlation with nearby records within 500 km over the last 500 years and a longer record length. Thus, we avoid involving too much local climate information which would decrease the spatial correlation length as well as overweighting regions with many proxies compared to regions that contain only sparse proxy information.

After the outlined procedure, 29 TRW and eight MXD chronologies are kept. Non-tree-ring proxy records were selected based on published articles, which have been used by Ge *et al*.^26^ or Ljungqvist *et al*.^43^ (see Table S1). Together with one tree-ring isotope record, twelve historical documentary series, four ice cores, three speleothems, three lake sediments, and one marine record, we have in total sixty-one warm-season temperature-sensitive proxy data from different regions over East Asia (Table S1). Their locations are shown in Fig. 1. Here, we acknowledge a potential uncertainty generated from different optimal seasons some proxy records have.

After the selection, all of the proxy records at annual resolution were averaged to decadal scale. In this study, we reconstructed decadal-resolution temperature rather than annual-resolution temperature as our proxy records with annual resolution are largely restricted to the western mountainous area or high-latitude areas.

### Global climate model (GCM) simulation data

We adopted the eleven millennium-length simulations performed with nine state-of-the-art GCM following the PMIP3/CMIP5 standards^34,44^ (see Table S2). Their regional temperature simulations over different continents have been compared with reconstructions^35^. The geographical window used in this work to represent the area of East Asia is 60 °–160 ° E/10 °–60 ° N. The variable considered is the 2 m air temperature. The simulated monthly series have been prepared to mimic the resolution and seasonality of the reconstruction. Also, all simulations have been remapped to a common 5 ° × 5 ° grid for the spatial comparison.

### Bayesian hierarchical model (BHM)

In the frame of non-regression approaches, BHM interprets surface temperature anomalies as a first-order auto-regression (AR1) process in time with exponentially decaying spatial covariance. In this study, instrumental data were modeled as temperature anomalies with white noise; proxy records like documentary data were modeled as a linear function of temperature anomalies plus a white noise term; and proxy records like TRW which exhibit strong persistence^45^ were modeled in three terms: a linear function of temperature anomalies, a AR1 process term (describing that the tree ring growth in the present-year is associated with the one in the previous-year) and a white noise term. To test the robustness of BHM field reconstructions and the corresponding regional average temperature time series, we generated two ensembles of CFRs using the full proxy network (59 proxies) and a sub dataset (denoted here Frozen1000) including only 13 proxy records which cover the whole last millennium (see Table S1). Each ensemble contains 1000 members and each member is one CFR going back to the beginning of the Common Era. The CFR members were taken from the predictive run (without instrumental data as input), which enables the comparison between the reconstruction and the instrumental data. The regional average temperature reconstructions generated from the two ensembles generally agree with each other (see “Bayesian hierarchical model” section in the supplementary file for more about the method, its implementation and related results). The spatial mean time series for each ensemble member was computed by averaging area-weighted (weighted by multiplying the cosine of latitude) values in relevant grid boxes. The temperature anomaly estimation for the ensemble median was calculated in two ways: 1) the point-wise median ensemble of reconstructions and the 90 percentiles confidence level, which takes the 50^th^ percentile at each decade as the best estimate of temperature anomaly (the median) and the 5^th^ and 95^th^ percentiles produce 90% point-wise uncertainty envelopes; and 2) the path-wise median ensemble of reconstructions and the 90 percentiles confidence intervals, which are addressed in “*Deepest curve and path-wise confidence intervals”*.

### Composite method

The proxy data were first standardized individually, and then the average was calculated to present regional temperature variations. This method could sufficiently reproduce large-scale temperature variations out of small and heterogeneous datasets^27,46,47^.

### Deepest curve and path-wise confidence intervals

As proxy records and reconstructions normally have high autocorrelation^45,48^, we considered each ensemble member as a function generated by continuous smooth dynamics. We drew information from a set of functional data by applying function data analysis. As an important concept to sort functional data, data depth introduces a measure to rank a sample of function data from the center outwards. In general, each ensemble member was drawn as a continuous curve. Then we computed the depth values of all the curves and ranked the curves by decreasing depth values following a modified half region depth approach^49^, which defines band depth by taking a graph-based approach.

Each ensemble member was considered as a function *y*_i_(*t*)*, i* = 1, …, 1000, generated by the stochastic process *Y*. The depth of *y* was measured as

1 $$H(y)=min\{SL(y),IL(y)\}.$$

*SL*, the superior lengths, indicates the “proportion of time” that the stochastic process *Y* is greater than y; and *IL*, the inferior length, indicates the “proportion of time” that the stochastic process *Y* is smaller than y. They can be calculated in the following manners:

$$SL(y)=\frac{1}{n\lambda (I)}\sum _{i=1}^{n}\lambda \{t\in I:y(t)\le {y}_{i}(t)\},$$

2 $$IL(y)=\frac{1}{n\lambda (I)}\sum _{i=1}^{n}\lambda \{t\in I:y(t)\ge {y}_{i}(t)\}$$

where $\lambda$ stands for the Lebesgue measure on **R**.

Then *y*_i_(*t*)*, i* = 1, …, 1000 can be ranked as y_[1]_(*t*), …, *y*_[1000]_(*t*) according to decreasing values of *H*(*y*_*i*_), where y_[1]_(*t*) denotes the deepest curve or simply the median curve and *y*_[1000]_(*t*) the most outlying curve. The deepest curve has the shortest distance to the mean. We used this concept to find the most central ensemble member *y*_[1]_(*t*) and let it represent the median trajectory of reconstructed temperature variations. The upper and lower boundaries of the band that is bounded by the curves *y*_[1]_(*t*), …, *y*_[900]_(*t*) are used as path-wise 90% confidence intervals. Estimating uncertainties in a path-wise manner enabled us to judge whether one event is an outlier from the perspective of the whole time period rather than at one specific time interval. The concept of path-wise uncertainties was adopted in former paleo-climate reconstructions^19,50^.

### Robustness test of the regional average temperature reconstruction

We applied two strategies to generate different regional average temperature reconstructions. The first one is to use the same data network (the full data network) but two different methods (BHM and composite method, the particularities of both methodologies has been briefly addressed above and more details can be found in the supplementary file). The second approach makes use of the same method, BHM, but two different data networks (the full data network versus “Frozen1000”). The four regional average temperature reconstructions generated as explained before are shown in Figs S2, S3. Correlation analysis among the four reconstructions shows that they are highly correlated with each other (*r* > 0.86, *p* < 0.01). They all indicate a multi-century warm period around 900–1200 CE followed by a multi-century long cool period around 1450–1850 CE and a continuous warming tendency after the 1850s decade with the modern warming during the second half of 20^th^ century. The temperature in the 1990s is seen noticeably higher than any previous warm conditions.

### Gridded temperature verification

The null hypothesis, *H*_0_, states that the observed temperature anomaly is well “predicted” by the reconstructions against the alternative hypothesis, *H*_*A*_, that the observed temperature anomaly is far different from the reconstructions. The null hypothesis is tested for each decade at each grid point for the period 1921–2000 CE. Let *T* denotes the reconstructed temperature and *T*_CRU_ the observed temperature, assuming that the observed record only falls at the tail of the empirical distribution of the 1000 ensemble members. The corresponding *P* value is calculated as following^51^, which indicates the proportion of reconstructed temperature anomalies which are more extreme than the observed temperature anomalies.

3 $$p=2\,\min (\frac{1}{1000}\sum _{i=1}^{1000}I({T}_{i} < {T}_{CRU}),\frac{1}{1000}\sum _{i=1}^{1000}I({T}_{i} > {T}_{CRU}))$$

When this *P* value is sufficiently small, we reject *H*_0_. Then we follow the false discovery rate procedure^52,53^ to reject *H*_0_ at all locations *i* for which *p*_*i*_ ≤ *p*_*k*_, where

4 $$k=\mathop{\max }\limits_{i=1,\mathrm{..}.,n}\{i:{p}_{[i]}\le q\frac{i}{n}\}$$

with *p*_*[i]*_*, i* = *1*, …, *n* are ranked p-values in an ascending order.

The field reconstruction has been verified against the instrumental record at each grid point and for each decade during 1921–2000 CE. Only for a few grid cells, the reconstruction is statistically significantly different from the instrumental temperature target (Fig. S4).

## Electronic supplementary material

supplementary file
