# Supplementary material for: East Asian warm season temperature variations over the past two millennia
Source: Sci Rep. 2018 May 16;8:7702. doi: 10.1038/s41598-018-26038-8 (PMC5955927; doi:10.1038/s41598-018-26038-8)
Supplement: Supplementary file 1 — supplementary file [file 41598_2018_26038_MOESM1_ESM.docx]

**Supplementary Information**

**East Asian warm season temperature variations over the past two millennia**

Huan Zhang^1^, Johannes P. Werner^2^, Elena García-Bustamante^3^, Fidel González-Rouco^4^, Sebastian Wagner^5^, Eduardo Zorita^5^, Klaus Fraedrich^6^, Johann H. Jungclaus^6^, Fredrik Charpentier Ljungqvist^7,8^, Xiuhua Zhu^9^, Elena Xoplaki^1^, Fahu Chen^10^, Jianping Duan^11^, Quansheng Ge^12^, Zhixin Hao^12^, Martin Ivanov^1^, Lea Schneider^1^, Stefanie Talento^1^, Jianglin Wang^13^, Bao Yang^13^, Jürg Luterbacher^1,14^,

1 Department of Geography, Climatology, Climate Dynamics and Climate Change, Justus Liebig University Giessen, Giessen, Germany

2 University of Bergen, Department of Earth Science and Bjerknes Centre for Climate Research, Allégt. 41, NO-5020 Bergen, Norway

3 Department of Energy, Renewable Energy Section, Research Center for Energy, Environment and Technology (CIEMAT), Madrid, Spain

4 Department of Physics of the Earth and Astrophysics,  IGEO (UCM-CSIC), Universidad Complutense de Madrid, Madrid, Spain

5 Institute for Coastal Research, Helmholtz-Zentrum Geesthacht, D-21502 Geesthacht, Germany

6 Max-Planck Institute for Meteorology, Bundesstrasse 53, D-20146 Hamburg, Germany

7 Department of History, Stockholm University, Stockholm, Sweden

8 Bolin Centre for Climate Research, Stockholm University, Stockholm, Sweden

9 Center for Earth System Research and Sustainability, CliSAP, University of Hamburg, Hamburg, Germany

10 MOE Key Laboratory of Western China’s Environmental System, Lanzhou University, Lanzhou 730000, China

11 State Key Laboratory of Vegetation and Environmental Change, Institute of Botany, Chinese Academy of Sciences, 100093 Beijing, China

12 Institute of Geographic Sciences and Natural Resources Research, Chinese Academy of Sciences, 11A, Datun Road, Chaoyang District, Beijing 100101, People’s Republic of China

13 Key Laboratory of Desert and Desertification, Northwest Institute of Eco-Environment and Resources, Chinese Academy of Sciences, Lanzhou, China

14 Centre for International Development and Environmental Research, Justus Liebig University Giessen, Giessen, Germany

******Corresponding author email: huan.zhang*@geogr.uni-giessen.de

*1. Reconstructions based on composite method*

The reconstructions on the basis of the composite method applied over the two different proxy data network (12 and 59 proxies respectively) are shown in Fig. S2. The two composite reconstructions have a good agreement in the evolution of temperature variations.

*2. Bayesian hierarchical model (BHM)*

*2.1 Model introduction*

BHM[[1]](#endnote-1)-[[2]](#endnote-2) is characterized by a process level and a data level. The process level here describes the evolution of the average warm-season temperature anomaly field, T, as

$T_{t+1}-u=a(T_{t}-u)+\varepsilon_{T,t}$ (1)

where $u$ is the field mean, $a$ is the coefficient of an AR1 process, the subscript t indexes time. $\varepsilon_{T,t}$ is the inter-decadal spatial-temporal variability of the temperature field with an exponentially decaying spatial correlation$\varepsilon_{T,t}$~$N(0,\sum)$,.

In the data level, the instrumental observations $\text{W}_{I,t}$ are modeled as noise version of the true anomalies:

$\text{W}_{I,t}=\text{H}_{I,t}(\text{T}_{t}+\varepsilon_{I,t})$ (2)

where $\text{H}_{I,t}$ is a selection matrix of zeros and ones, which picks out the locations of instrumental data at each decade and$\varepsilon_{I,t}$ is the noise terms with a multivariate normal distribution as$\varepsilon_{I,t}$~ $N(0, \text{I }\tau_{I}^{2})$, $\text{I}$denotes the identity matrix.

The MXD and documentary data are assumed to have a linear relationship with the true temperature

$\text{W}_{P_{1},t}=\text{H}_{P_{1},t}(\beta_{1,P_{1}}\text{T}_{t}+\beta_{0,P_{1}}+\varepsilon_{P_{1},t})$ (3a)

where$\beta_{1}$ and $\beta_{0}$ are the slope and intercept terms, $\text{H}_{P_{1},t}$ indicates the locations of proxy data available in space at time *t*, and $\varepsilon_{P_{1},t}$is the noise terms for proxy data with a distribution as $\varepsilon_{P,t}$~$N(0, \text{I }\tau_{P}^{2})$.

For the proxy types TRW, ice cores, speleothems, lake sediments and marine data, an extra memory term is included, to present the current year’s tree-ring width growth which is influenced by the former year’s climate conditions through biological processes[[3]](#endnote-3)-[[4]](#endnote-4)[[5]](#endnote-5).

$\text{W}_{P_{2},t}=\text{H}_{P_{t},t}(\beta_{2,P_{2}}\text{W}_{P_{2},t-1}+\beta_{1,P_{2}}\text{T}_{t}+\beta_{0,P_{2}}+\varepsilon_{P_{2},t})$ (3b)

Where $\beta_{2}$ indicates the memory strength of this proxy type.

*2.2 Implementation*

For the purpose of comparing the reconstruction with instrumental data, the CFRs were generated after two steps according to Luterbacher et al and Gelman et al[[6]](#endnote-6). In the first step, BHM was run with both instrumental data and proxy records included for 3000 iterations of Gibbs sampler. The BHM was run using three chains of the Gibbs samplers with different initial values. After 500 samples, all parameters converged and their posterior distributions from last 1000 samplings agreed with each other among all three chains. To avoid divergence in earlier decades when only few proxy data are available, the inference run was done for the period 800-2000 CE only. In the second step, also termed a “prediction run”, BHM was run for the whole period (1-2000CE) with only the proxy records included and instrumental data excluded. The temperature field was updated while sampling the posterior distribution from the first step. In this study, we only use the last 1000 iterations of the Gibbs sampler from the prediction run.

For the reconstructions using the full proxy network, documents-based data were standardized by removing a common mean and scaling by a common standard deviation with respect to the period of 800-2000 CE. Tree-ring, ice core, lake sediment, marine and speleothem proxies were normalized individually with respect to the period 800-2000 CE. Each proxy group shared a common set of parameters ($\beta_{0},\beta_{1},\tau_{p}$ and/-$\beta_{2}$). For reconstructions using 12 millennium-length proxy data network, each single proxy was standardized individually with respect to 1000-2000 CE, and was given a distinct set of parameters. CRU data were standardized by removing a common mean and scaling by a common standard deviation with respect to the period of 1920-2000 CE, later the reconstructions were rescaled with mean and standard variance of CRU data in the period 1921-2000 CE.

*2.3 Comparison between BHM and BHM12*

Figure S3 shows the regional average temperature variations which are generated using BHM with the full dataset (59 proxies) and the reduced dataset (12 proxies) respectively (hereafter BHM-median and BHM12). Along with the point-wise median values, 90% point-wise and path-wise confidence intervals are also given. Generally they show considerable similarity, and the correlation between the two reconstructed regional temperature time series reaches up to *r*=0.86, (*p*=0.01). The variation of temperature of BHM is slightly higher compared to the BHM12 in the last 1200 years. BHM12 and BHM have a general good agreement with the characteristics of the composite reconstructions (Fig. S2).

*2.4 Analysis of the probability distribution parameters in BHM reconstructions*

Fig. S5 presents the posterior distributions of all the scalar parameters that define BHM. The posteriors are strongly influenced by the data as they are largely different from their prior distributions, which are flat. The posterior distribution of the AR(1) coefficient $\alpha$has its most mass between 0.8 and 0.82. Posterior draws of the $\alpha$ when only including the 132 CRU instrumental decadal-mean time series are centered around 0.56. This value is still higher than the detected autocorrelation strength of annual temperature in CRU data. Two factors probably increase the autocorrelation in the data: decadal scale variations of temperature are less influenced by small-scale stochastic processes but rather by the strong trends in the instrumental period. The further increase from 0.56 to 0.8 of the $\alpha$ when the proxy data are included in BHM can likely be related to the memory in different proxy data, especially tree-ring proxies.

The posterior distribution of spatial correlation length has its most mass between 714 and 833 km. This value is smaller than the correlation length found in Arctic[[7]](#endnote-7) (ca. 2000km) and Europe (ca. 2000km). As proxies from western part of East Asia are mainly collected in the mountainous area around Tibet, these proxies probably explain only very local climate.

The posterior distributions of the $\beta_{2}(k)$, $\beta_{1}(k)$, $\beta_{0}(k)$, and $\tau^{2}(k)$ , k = 1,2, . .. ,7, offer insight into the relative strengths of the different proxy data types. The distributions of the $\tau^{2}(k)$, the observational error variance for each proxy type, indicate that the tree ring isotope features the smallest error, and tree ring density the largest error and tree ring width the second largest. The parameter of $\beta_{1}(k)$ indicates the weighting of each proxy type when they are combined to infer the field, with the tree ring isotope featuring the highest values, and the tree ring width and tree ring density the lowest values. Even though tree-ring proxies are one major data resource for our reconstructions, the very low values of $\beta_{1}(k)$ and the high value $\tau^{2}(k)$ for the tree ring width and tree ring density records indicate that they are less informative of the decadal-mean temperature. Low-frequency climate information provided by the proxies within these two tree-ring types is probably corrupted by the segment length curse[[8]](#endnote-8) and detrending procedures[[9]](#endnote-9). Note, the summer temperature field reconstruction on the basis of pure tree-ring proxies by Cook et al[[10]](#endnote-10) was pointed out as having “substantial uncertainties in low frequencies” by PAGES 2K Consortium[[11]](#endnote-11). Besides, tree-ring width data also suffer from relative high autocorrelation, see the distribution of $\beta_{2}(k)$ for tree-ring width.

*Comparison with Cook2013 and Shi2015 on the spatial temperature differences*

BHM, Cook2013 and Shi2015 are compared on the spatial temperature differences for the transitions: MCA (900–1200 CE) - LIA (1450–1850 CE), present-day (1950–2000 CE) - MCA and present-day - LIA (Fig. S8). Even though Shi2015 and Cook2013 share a significant fraction of the variation of the regional average temperature (*r*=0.79, *n*=108, *p*<0.01), Cook2013 often suggests different climate transition patterns from both Shi2015 and BHM over eastern China. BHM and Shi2015 have generally better agreement on temperature spatial patterns during the climate transitions, but the two differ regarding whether the Tibetan Plateau experienced warmer climate over the MCA comparing to present day.

**Figure legends**

Figure S1. Area-weighted warm-season (MJJAS) temperature anomalies (1961-1990 climatology) over East Asia using the raw CRU4v data (black line) and filled CRU4v data using the RegEM-Ridge algorithm (green line), and decadal mean temperature (read line)

Figure S2. Relative changes of temperature reconstructions derived directly averaging 12 millennium length proxy records and all 59 proxy records which are first standardized according to the period 1001-2000 CE

Figure S3. Reconstructed area-weighted decadal temperature anomalies (with respect to 1500-1850 CE) using Bayesian hierarchical model with full proxy network (blue) and Frozen 1000 network (12 proxy data, red) respectively. Solid line: the median values; shades: 90% point-wise confidence intervals; dashed line: 90% path-wise confidence intervals.

Figure S4. Maps of *P* values (the proportion of reconstructed temperature anomalies which are more extreme than the observed temperature anomalies) and the percentage of grid cells where *P>P* _k_ (the observed temperature anomaly is well “predicted” by the reconstructions). Cross marker: *P*< *P* _k_ (the reconstructions is statistically significantly different from the observed temperature anomaly). The figure was generated using Matlab 2015b. The map in the figure was queried from Google Static Map APIs (http://code.google.com/apis/maps/).

Figure S5. (a) Posterior distributions of the scalar parameters that define Bayesian hierarchical model using 59 proxy records. (b) The distributions of the proxy parameters are shown for each of the seven types of proxy.

Figure S6: Reconstructed China/ East Asia decadal temperatures anomalies (solid lines, w. r. t. 1500-1850) with associated uncertainty range (shades) covering the post 850 CE period. All series are smoothed with a 31-yr moving average. Dashed black line: 90% path-wise confidence intervals of BHMs.

Figure S7. Reconstructed temperature differences for three periods: MCA (900-1200) minus LIA (1450-1850); present-day (1950-2000) minus MCA; and present-day minus LIA. The figure was generated using Matlab 2015b. The map in the figure was queried from Google Static Map APIs (http://code.google.com/apis/maps/).

Figure S8. The BHM-median temperature anomalies (w.r.t. 1961-1990 CE, red) and the reconstructed solar irradiance by Vieira et al. [[12]](#endnote-12) (blue).

Table S1. Proxy paleo-temperature sensitive records used in this study

Table S2. Models and experiments considered for the analysis (column 1); horizontal and vertical resolution of atmospheric and ocean model components (columns 2 and 3); set of external forcings implemented within each simulation (column 4); number of ensemble members and length (column 5) and original reference describing the experiments (column 6).


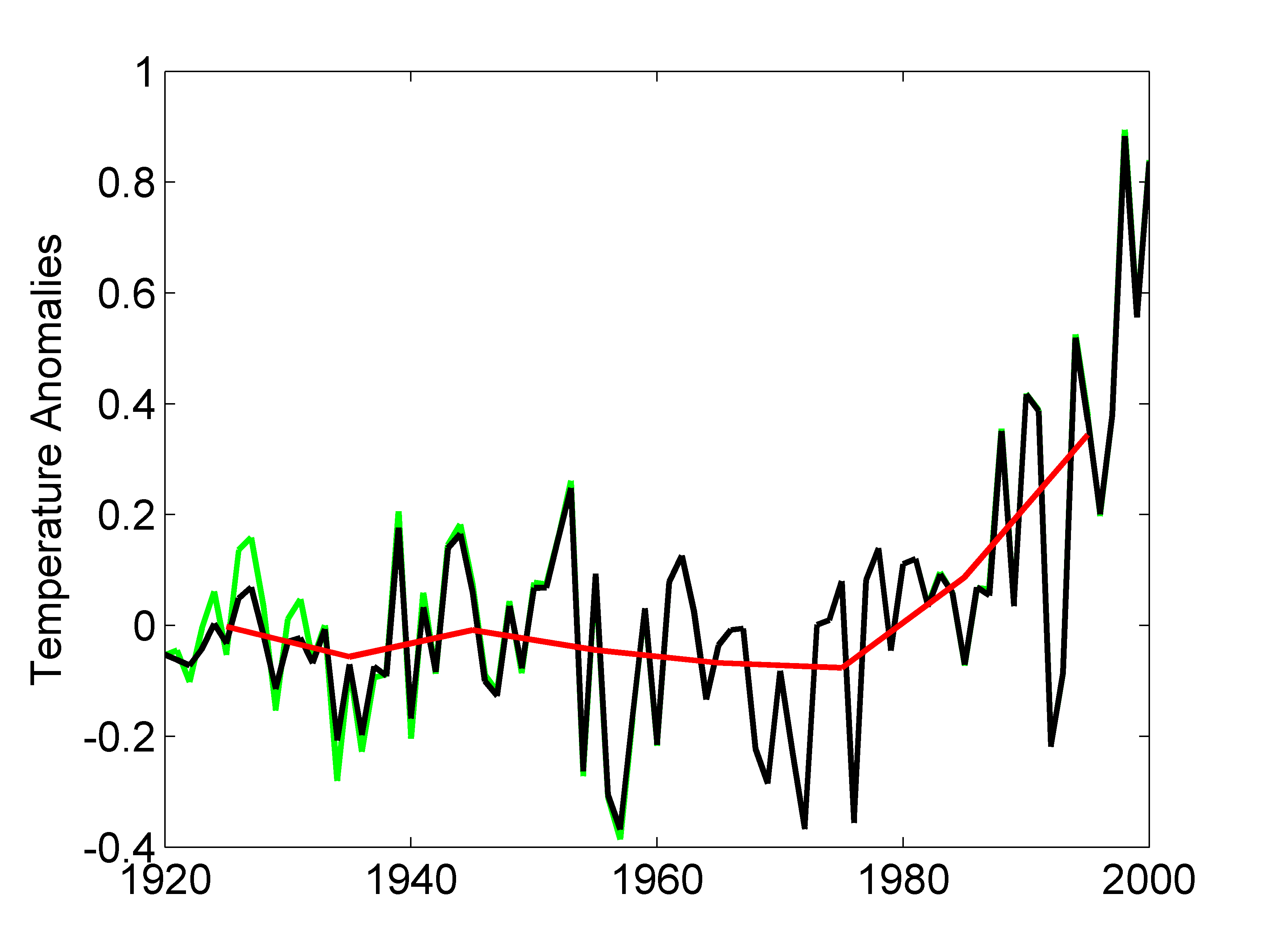


Figure S1


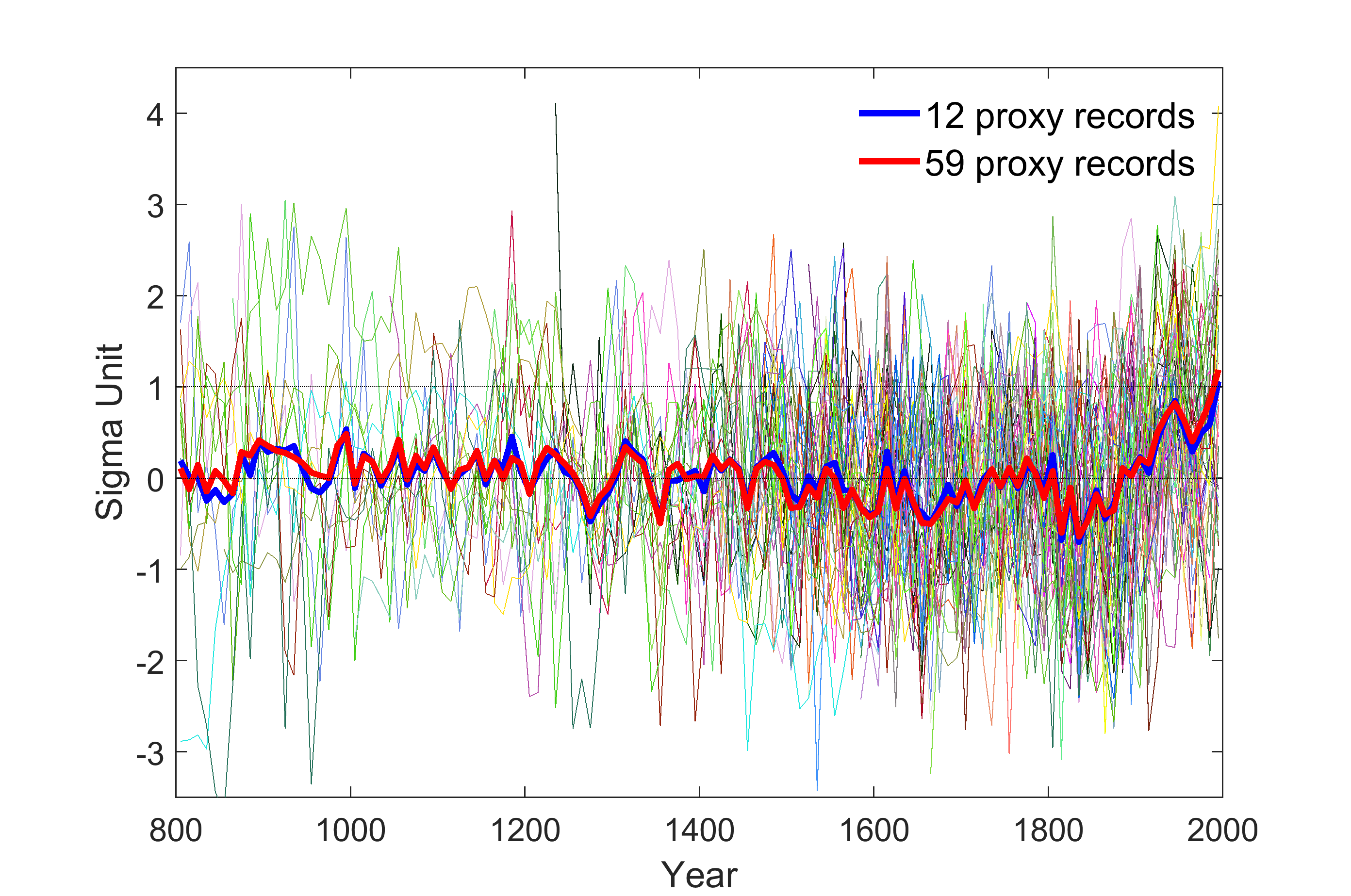


Figure S2


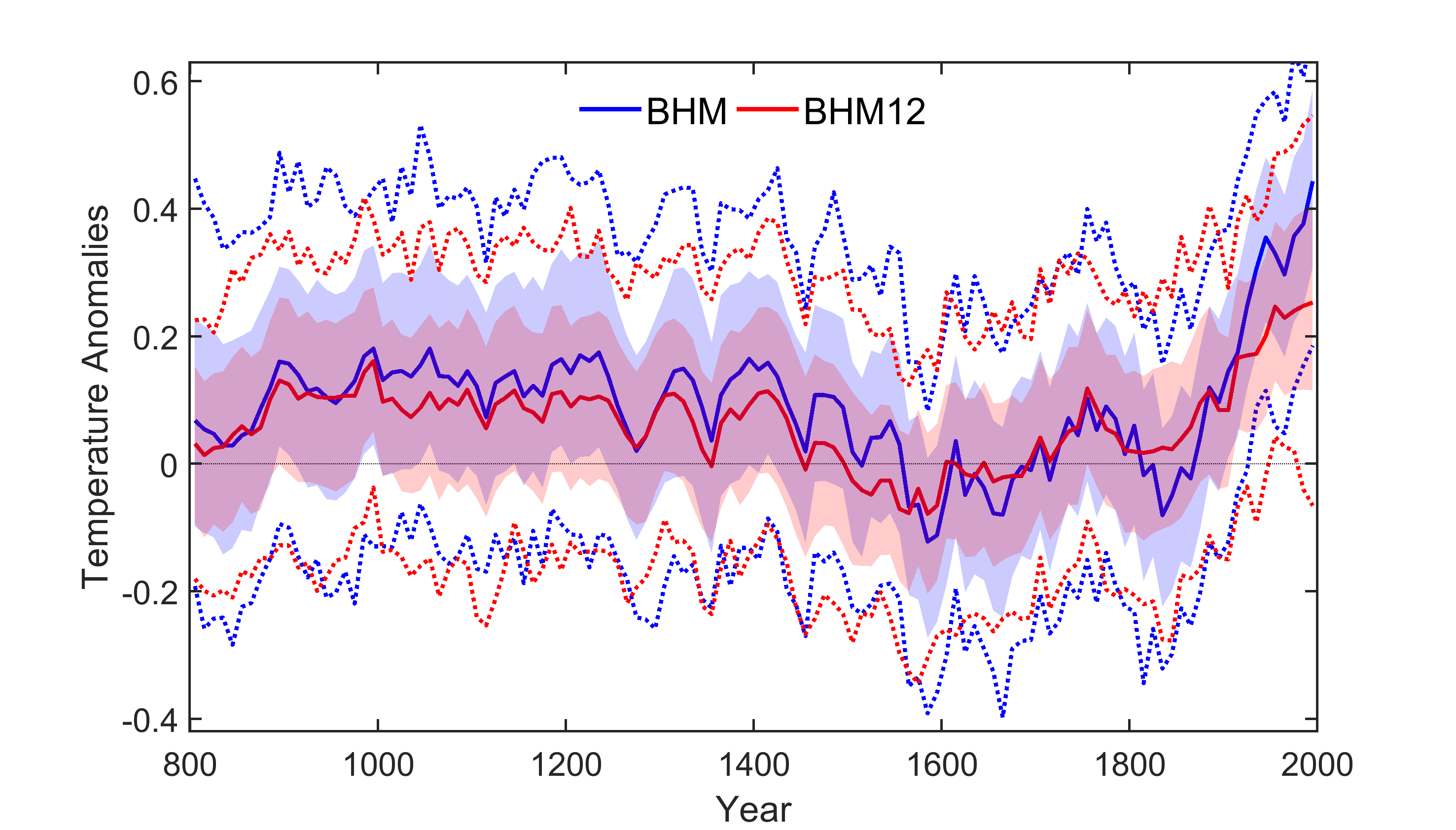


Figure S3


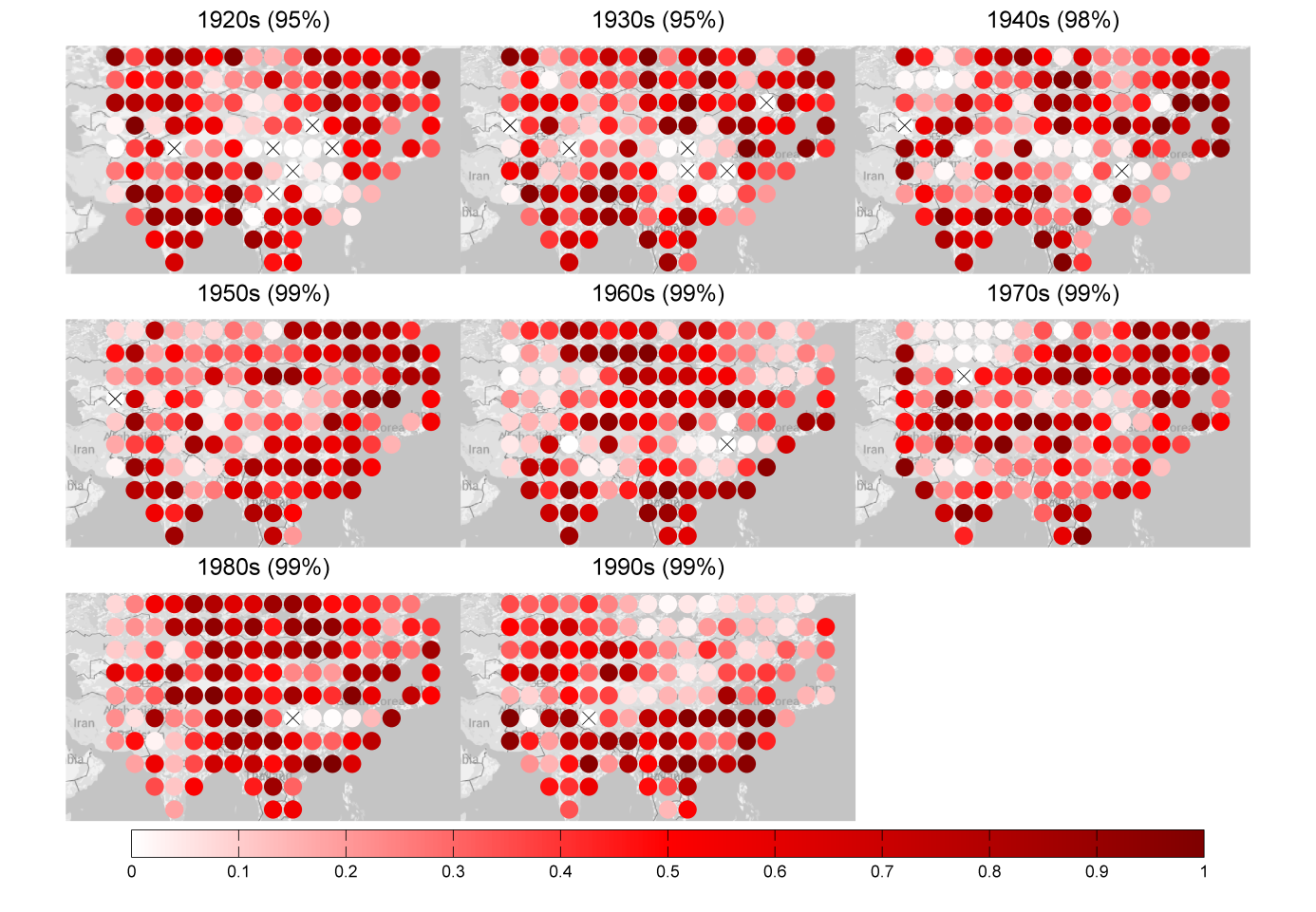


Figure S4


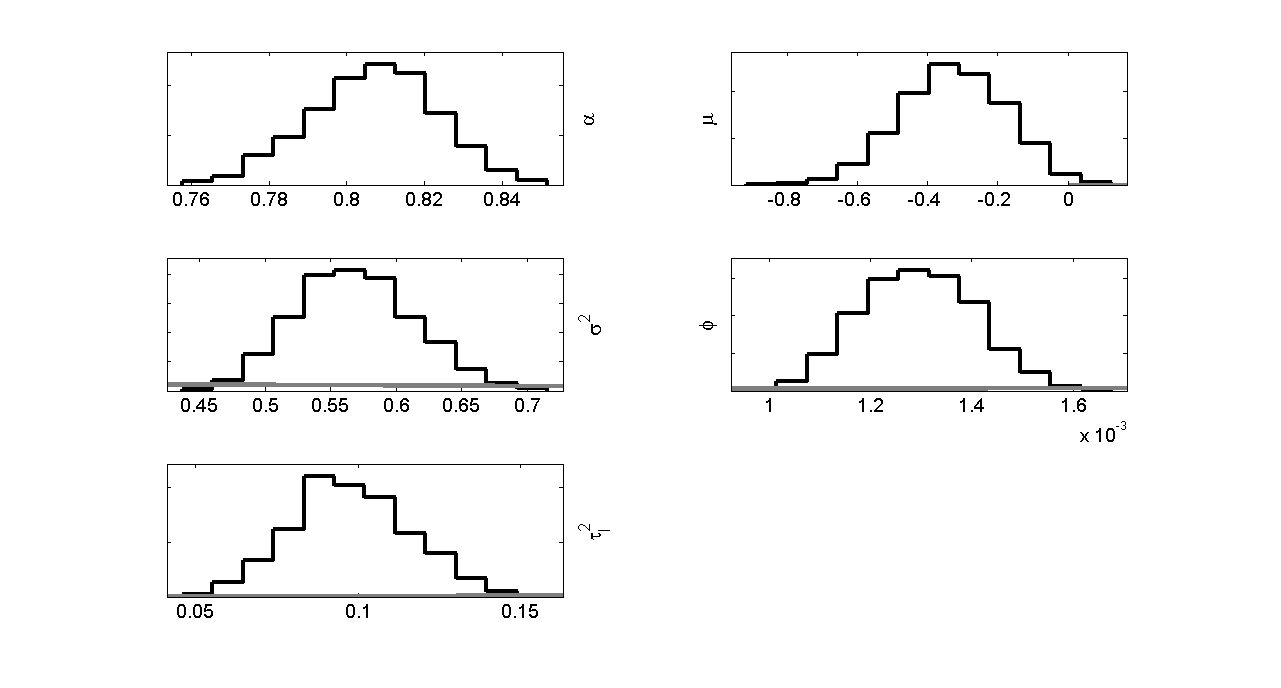


(a)


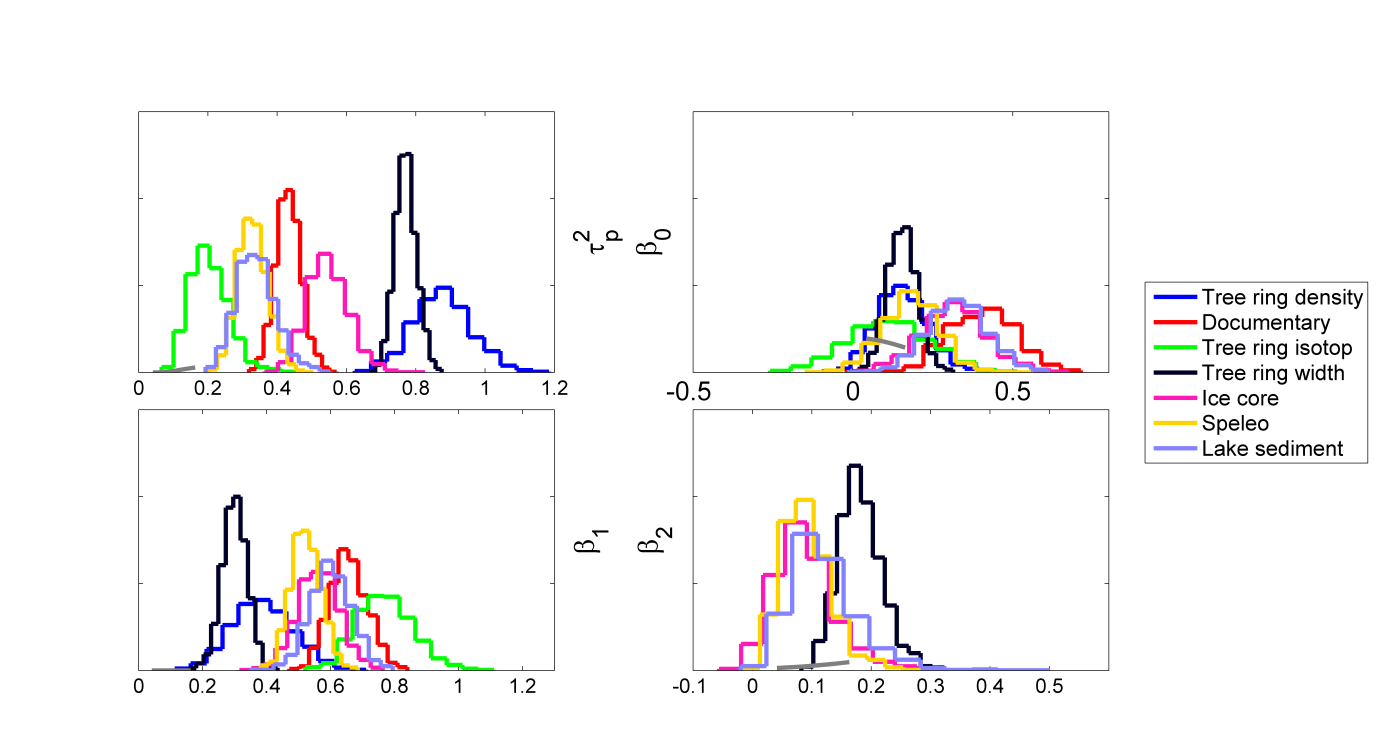


(b)

Figure S5

***
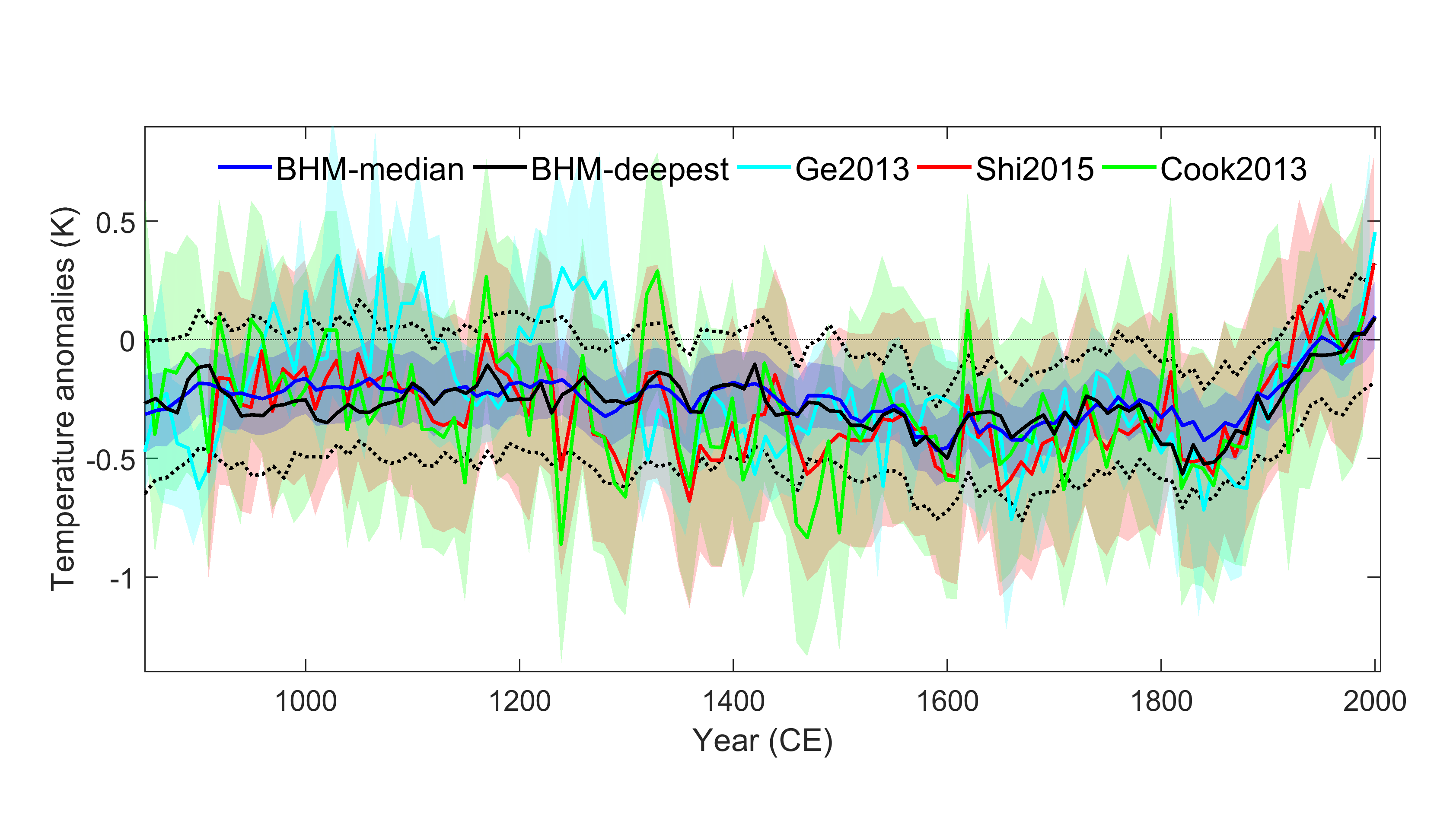
***

Figure S6

***
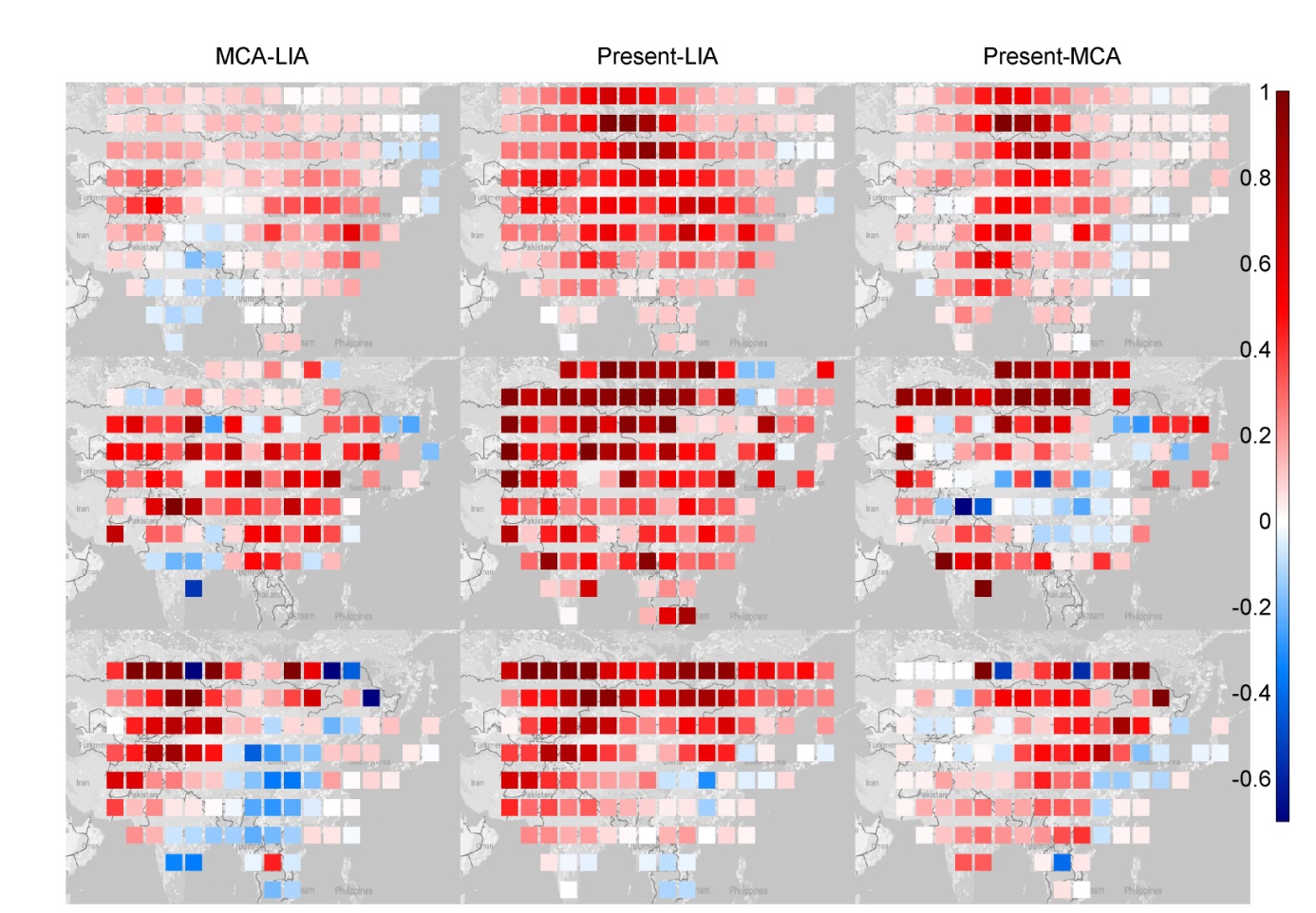
***

**Shi2015**

**BHM**

**Temperature difference (°C)**

**Cook2012**

Figure S7


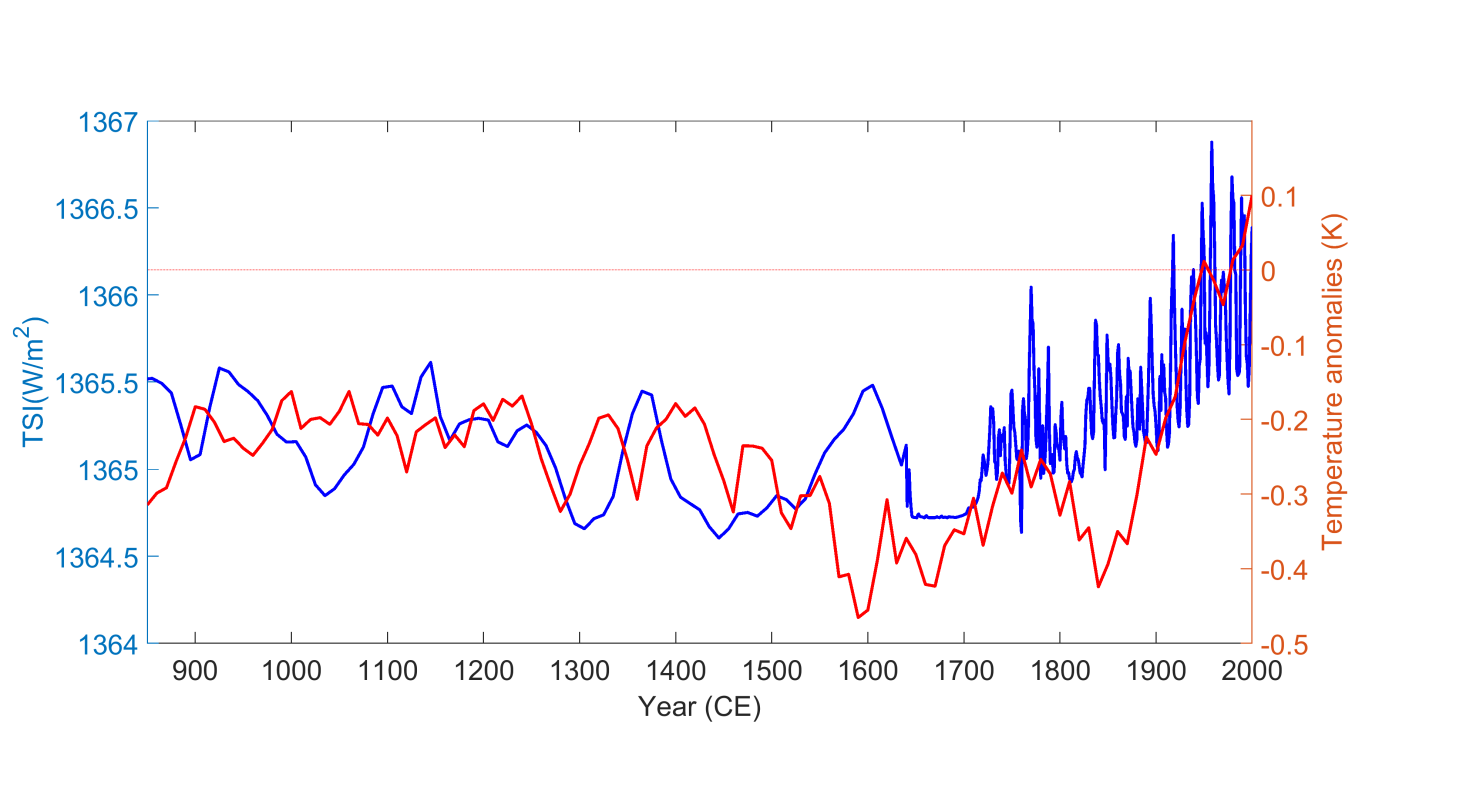


Figure S8

Table S1

| Group | Lon./Lat. | Archive | Prox Type | Period | Res | Reference |
| --- | --- | --- | --- | --- | --- | --- |
| 1 | 88.1°E /49.62°N | Tree ring | TRW | 1561-2000 | A |  |
| 1 | 85.23°E /50.87°N | Tree ring | TRW | 1568-1995 | A |  |
| 1 | 87.83°E /50.17°N | Tree ring | TRW | 1418-1999 | A |  |
| 1 | 87.97°E /50.68°N | Tree ring | TRW | 1555-2000 | A |  |
| 1 | 87.65°E /50.48°N | Tree ring | TRW | 1581-1994 | A |  |
| 1 | 85.37°E /50.15°N | Tree ring | TRW | 1581-1994 | A |  |
| 1 | 90.97°E /27.42°N | Tree ring | TRW | 1457-2000 | A |  |
| 1 | 89.75°E /27.95°N | Tree ring | TRW | 1453-2000 | A |  |
| 1 | 101.53°E /37.93°N | Tree ring | TRW | 1288-2000 | A |  |
| 1 | 100.07°E /35°N | Tree ring | TRW | 1433-2000 | A |  |
| 1 | 98.4°E /27.88°N | Tree ring | TRW | 1530-2000 | A |  |
| 1* | 130.5°E/ 30.33°N | Tree ring | TRW | 1-1999 | A |  |
| 1 | 72.58°E /40.17°N | Tree ring | TRW | 1157-1995 | A |  |
| 1* | 71.5°E/39.83°N | Tree ring | TRW | 870-1995 | A |  |
| 1 | 78.97°E /42.42°N | Tree ring | TRW | 1551-2000 | A |  |
| 1 | 79.45°E /42.15°N | Tree ring | TRW | 1528-2000 | A |  |
| 1 | 90.97°E /47.1°N | Tree ring | TRW | 1375-2000 | A |  |
| 1 | 91.57°E /49.92°N | Tree ring | TRW | 1326-1998 | A |  |
| 1 | 86.45°E /27.7°N | Tree ring | TRW | 1417-1998 | A |  |
| 1 | 82.03°E /29.52°N | Tree ring | TRW | 1566-1997 | A |  |
| 1 | 88.02°E /27.5°N | Tree ring | TRW | 1525-1999 | A |  |
| 1 | 87.2°E /27.73°N | Tree ring | TRW | 1509-1999 | A |  |
| 1 | 74.8°E /35.33°N | Tree ring | TRW | 1317-2000 | A |  |
| 1 | 74.58°E /36.03°N | Tree ring | TRW | 1240-1993 | A |  |
| 1 | 74.18°E /36.15°N | Tree ring | TRW | 1387-2000 | A |  |
| 1* | 75.5°E/35.17°N | Tree ring | TRW | 388-1993 | A |  |
| 1 | 98.87°E /31.95°N | Tree ring | TRW | 1519-1996 | A |  |
| 1 | 93.95°E /29.7°N | Tree ring | TRW | 1047-1993 | A |  |
| 1* | 98.93°E/48.3°N | Tree ring | TRW | 262-1999 | A | 10 |
| 2 | 75.92°E /34.25°N | Tree ring | MXD | 1777-1981 | A | [[13]](#endnote-13) |
| 2 | 75.47°E /34.65°N | Tree ring | MXD | 1682-1981 | A |  |
| 2 | 86.08°E /27.75°N | Tree ring | MXD | 1722-1978 | A | [[14]](#endnote-14) |
| 2 | 58.88°E /54.88°N | Tree ring | MXD | 1671-1993 | A |  |
| 2 | 106.88°E /53.18°N | Tree ring | MXD | 1687-1996 | A |  |
| 2 | 109.5°E /55.25°N | Tree ring | MXD | 1692-1996 | A |  |
| 2 | 97.5°E /27.5°N | Tree ring | MXD | 1453-1994 | A |  |
| 2 | 87.5°E /52.5°N | Tree ring | MXD | 1581-1994 | A |  |
| 3* | 74.99°E/36.37°N | Tree ring | $\delta^{13}C$ | 828-1998 | A | [[15]](#endnote-15) |
| 4 | 117.2°E /31.82°N | Historical documents | - | 1736-1991 | A | [[16]](#endnote-16) |
| 4 | 117.5°E /30°N | Historical documents | - | 1470-1970 | D | [[17]](#endnote-17) |
| 4 | 118°E /40°N | Historical documents | - | 1380-1990 | D | [[18]](#endnote-18) |
| 4 | 120°E /34°N | Historical documents | - | 1380-1990 | D |  |
| 4 | 113°E /29°N | Historical documents | - | 1470-1990 | D |  |
| 4* | 121°E/32°N | Historical documents | - | 994-1998 | D | [[19]](#endnote-19) |
| 4 | 114.5°E /30.5°N | Historical documents | - | 1470-1960 | D | [[20]](#endnote-20) |
| 4 | 112.5°E /23.5°N | Historical documents | - | 1470-1960 | D |  |
| 4 | 118°E /25°N | Historical documents | - | 1470-1960 | D |  |
| 4 | 113.23°E /23.16°N | Historical documents | - | 1400-1940 | D | [[21]](#endnote-21) |
| 4 | 117°E /36.63°N | Historical documents | - | 1470-1980 | D | [[22]](#endnote-22) |
| 4 | 118.8°E /32.1°N | Historical documents | - | 1470-1960 | D |  |
| 5 | 85°E /28°N | Ice core | $\delta^{18}O$ | 1010-1997 | D | [[23]](#endnote-23) |
| 5* | 96.4°E/38.1°N | Ice core | $\delta^{18}O$ | 795-1985 | D | [[24]](#endnote-24) |
| 5 | 81.29°E /35.17°N | Ice core | $\delta^{18}O$ | 1009-1989 | D |  |
| 5* | 89.05°E/33.55°N | Ice core | $\delta^{18}O$ | 1-2000 | D | [[25]](#endnote-25) |
| 6* | 109.05°E/33.4°N | Speleo | $\delta^{18}O$ | 799-1996 | D | [[26]](#endnote-26) |
| 6* | 115.56°E/39.47°N | Speleo | Lamina thickness | 800-1985 | A | [[27]](#endnote-27) |
| 7* | 102.33°E/34.24°N | Lake sediments | Alkenone | 799-1963 | D | [[28]](#endnote-28) |
| 7 | 100.5°E /36.6°N | Lake sediments | Rb/Sr ratio | 1050-2000 | D | [[29]](#endnote-29) |
| 7* | 87.61°E/51.76°N | Lake sediments | X-ray/ Rb/Sr ratio | 800-1999 | A | [[30]](#endnote-30) |

A=annual resolution; D=decadal resolution. The data with * are used for Frozen1000.

Table S2

| Model | Atmosphere | Ocean | Forcings | Simulations | Reference |
| --- | --- | --- | --- | --- | --- |
| CCSM4 | 0.9°×1.25°, L26 | Nominal 1°, L60 | [[31]](#endnote-31)-[[32]](#endnote-32) | (1) 850-1850 CE | [[33]](#endnote-33) |
| CSIRO-Mk3L-1-2 | 5.63°×3.21°, L18 | 2.81°×1.61°, L21 | - | (1) 851- 1850 CE | [[34]](#endnote-34) |
| FGOALS | 72×40L26 | 360×170L30 | SVG | (1) 1000-1999 CE | [[35]](#endnote-35) |
| GISS-E2-R | 2°×2.5°, L40 | 1°×1.25°, L32 | - | (3) 850-1850 CE | [[36]](#endnote-36) |
| HadCM3 | 3.75°×2.46°,L19 | 1.25°×1.25°, L20 | - | (1) 800- 2000 CE | [[37]](#endnote-37) |
| IPSL-CM5A-LR | 3.75°×1.88°, L26 | 1.98°×1.21°, L32 | - | (1) 850-2005 CE | [[38]](#endnote-38) |
| CESM1.0 | 1.25° × 0.9° , L80 | 1.0º × 1.0º, L60 | - | (1) 850-2100 CE | [[39]](#endnote-39) |
| MPI-ESM-P | 1.84°×1.84°, L47 | Nominal 1.5°, L40 | - | (1) 850-2005 CE | [[40]](#endnote-40) |
| ECHAM5/MPIOM (COSMOS) | 3.75°×3.75°,L19 | Nominal 3°, L40 | - | (E1:5) 800-2005 CE | [[41]](#endnote-41) |
|  |  |  | - | (E2:3) 800-2005 CE |  |

External forcing configuration for FGOALS: (S) solar forcing; (V) volcanic activity; (G) greenhouse gases

**References**

1. 1. Tingley, M.P. & Huybers, P. A Bayesian algorithm for reconstructing climate anomalies in space and time. Part 1. Development and applications to paleoclimate reconstructions problems. *J. Climate* **23**, 2759-2781 (2010). [↑](#endnote-ref-1)
2. 2. Werner, J.P., Luterbacher, J. & Smerdon, J.E. A Pseudoproxy Evaluation of Bayesian Hierarchical Modelling and Canonical Correlation Analysis for Climate Field Reconstructions over Europe. *J.* *Climate* **26**, 851-867 (2013). [↑](#endnote-ref-2)
3. 3. Werner, J.P., Toreti, A. & Luterbacher J. Stochastic models for climate reconstructions -- how wrong is too wrong? *Nolta Proc.* **24**, 528-531 (2014). [↑](#endnote-ref-3)
4. 4. Zhang, H., Yuan, N., Esper, J., Werner, J.P., Xoplaki, E., Büntgen, U., Treydte, K. & Luterbacher, J. Modified climate with long term memory in tree ring proxies. *Environmental Research Letters* **10(8)**, 084020 (2015). [↑](#endnote-ref-4)
5. 5. Luterbacher, J., Werner, J.P., Smerdon, J.E., Fernández-Donado, L., González-Rouco, F.J., Barriopedro, D., Ljungqvist, F.C., Büntgen, U., Zorita, E., Wagner, S., Esper, J., et al. European summer temperatures since Roman times. *Environmental research letters* ***11*(2)**, 024001 (2016). [↑](#endnote-ref-5)
6. 6. Gelman, A. et al. *Bayesian Data Analysis*. Chapman Hall CRC. (2003) [↑](#endnote-ref-6)
7. 7. Tingley, M.P., & P. Huybers. Recent temperature extremes at high northern latitudes unprecedented in the past 600 years. *Nature* **496**, 201-205 (2013). [↑](#endnote-ref-7)
8. 8. Cook, E.R., Briffa, K.R., Meko, D.M., Graybill, D.A., & Funkhouser, G. The “segment length curse” in long tree-ring chronology development for palaeolclimatic studies. *The Holocene* **5**, 229-237 (1995). [↑](#endnote-ref-8)
9. 9. Esper, J., Cook, E. R & Schweingruber, F. H. Low-frequency signals in long tree-ring chronologies for reconstructing past temperature variability. *Science* **295**, 22502253 (2002). [↑](#endnote-ref-9)
10. 10. Cook, E. et al. Tree-ring reconstructed summer temperature anomalies for temperate East Asia since 800 C.E. *Clim. Dynam.* **41**, 11-12 (2013). [↑](#endnote-ref-10)
11. 11. PAGES 2K Consortium. Continental-scale temperature variability during the last two millennia. *Nat. Geosci.* **6**, 339-346 (2013). [↑](#endnote-ref-11)
12. . Vieira, L. E. A., Solanki, S. K., Krivova, N. A., & Usoskin, I.G. Evolution of the solar irradiance during the Holocene. *Astron. Astroph.*, 531, A6, 10.1051/0004-6361/201015843, (2011). [↑](#endnote-ref-12)
13. 13. Bhattacharyya, A., LaMarche, V. C. Jr., & Hughes, M. K. Tree-ring chronologies from Nepal. *Tree-Ring Bulletin* (1992). [↑](#endnote-ref-13)
14. 14. Briffa, K. R., Osborn, T. J., Schweingruber, F. H., Jones, P. D., Shiyatov, S. G., & Vaganov, E. A. Tree-ring width and density data around the Northern Hemisphere: Part 1, local and regional climate signals. *The Holocene* **12(6)**, 737-757, (2002). [↑](#endnote-ref-14)
15. 15. Treydte, K. S., Frank, D. C., Saurer, M., Helle, G., Schleser, G. H., & Esper, J. Impact of climate and CO2 on a millennium-long tree-ring carbon isotope record. *Geochimica et Cosmochimica Acta* **73(16)**, 4635-4647, (2009). [↑](#endnote-ref-15)
16. 16. Hao, Z. X., Zheng, J. Y., & Ge, Q. S. Historical analogues of the 2008 extreme snow event over Central and Southern China. *Clim. Res.* **50**, 161-170 (2011). [↑](#endnote-ref-16)
17. 17. Wang, R., Wang, S., & Fraedrich, K. An approach to reconstruction of temperature on a seasonal basis using historical documents from China. *International Journal of Climatology* **11(4)**, 381-392, (1991).

    [↑](#endnote-ref-17)
18. 18. Wang, S.W., Ye, J.L. & Gong, D.Y. Climate in China during the Little Ice Age (in Chinese). *Quat. Sci.* **25**, 54-62. (1998). [↑](#endnote-ref-18)
19. 19. Zhang, Q., Gemmer, M. & Chen, J. Climate changes and flood/drought risk in the Yangtze Delta, China, during the past millennium. *Quat. Int.* **176**, 62-69 (2008). [↑](#endnote-ref-19)
20. 20. Zhang, D. E. Winter temperature changes during the last 500 Years in South China. *Chinese Sci. Bull.* **6**, 497-500 (1980). [↑](#endnote-ref-20)
21. 21. Zheng, S. Z. Climate in the Little Ice Age and its effects in Guangdong, China. *Chinese Sci. Bull*. **27**, 302-304 (1982) (in Chinese). [↑](#endnote-ref-21)
22. 22. Zheng, J. Y. & Zheng, S. Z. An analysis on cold/warm and dry/wet in Shandong Province during historical times. *Acta Geographica Sinica* **48**, 348-357 (1993) (in Chinese). [↑](#endnote-ref-22)
23. 23. Thompson, L. G., Mosley-Thompson, E., Davis, M. E., Lin, P. N., Henderson, K., & Mashiotta, T. A. Tropical glacier and ice core evidence of climate change on annual to millennial time scales. *Climatic Change* **59**, 137-155 (2003). [↑](#endnote-ref-23)
24. 24. Thompson, L.G., Mosley-Thompson, E., Brecher, H., Davis, M., Leon, B., Les, D., Lin, P.N., Mashiotta, T., Mountain, K. Abrupt tropical climate change: Past and present. *Proceedings of the National Academy of Sciences of the United States of America* **103(28),** 10536-10543 (2006). [↑](#endnote-ref-24)
25. 25. Thompson, L.G., Yao, T., Davis, M.E., Mosley-Thompson, E., Mashiotta, T.A., Lin, P.N., Mikhalenko, V.N., & Zagorodnov, V.S. Holocene climate variability archived in the Puruogangri ice cap on the central Tibetan Plateau. *Annals of Glaciology* **43(1)**, 61-69 (2006). [↑](#endnote-ref-25)
26. 26. Paulsen, D., Li, H. & Ku, T. Climate variability in central China over the last 1270 years revealed by high-resolution stalagmite records. *Quat. Sci. Rev.* **22**, 691-701 (2003). [↑](#endnote-ref-26)
27. 27. Tan, M., Liu, T., Hou, J., Qin, X., Zhang, H. & Li, T. Cyclic rapid warming on centennial scale revealed by a 2650-year stalagmite record of warm season temperature. *Geophys. Res. Lett.* **30(12)**, 1617 (2003). [↑](#endnote-ref-27)
28. 28. He, Y., Zhao, C., Wang, Z., Wang, H., Song, M., Liu, W., Liu, Z. Late Holocene coupled

    moisture and temperature changes on the northern Tibetan Plateau. *Quat. Sci. Rev.* **80**, 47-57 (2013). [↑](#endnote-ref-28)
29. 29. Shen, J., Zhang, E. L., & Xia, W. L.: Records from lake sediments of the Qinghai Lake to mirror climatic and environmental changes of the past about 1000 year (in Chinese). *Quat. Sci.* **21**, 508-513 (2001). [↑](#endnote-ref-29)
30. 30. Kalugin, I. A., Daryin, A. V. & Babich, V. V. Reconstruction of annual air temperatures for three thousand years in Altai region by lithological and geochemical indicators in Teletskoe Lake sediments. *Dokl. Earth Sci.* **426**, 681-684 (2009). [↑](#endnote-ref-30)
31. 31. Schmidt, G.A., Jungclaus, J.H., Ammann, C.M., Bard, E., Braconnot, P.C.T.J.D.G., Crowley, T.J., Delaygue, G., Joos, F., Krivova, N.A., Muscheler, R., Otto-Bliesner, B., et al. Climate forcing reconstructions for use in PMIP simulations of the last millennium (v1. 0). *Geoscientific Model Development* **4(1)**, 33-45 (2011). [↑](#endnote-ref-31)
32. 32. Schmidt, G.A., Jungclaus, J.H., Ammann, C.M., Bard, E., Braconnot, P., Crowley, T.J., Delaygue, G., Joos, F., Krivova, N.A., Muscheler, R., Otto-Bliesner, B.L., et al. Climate forcing reconstructions for use in PMIP simulations of the Last Millennium (v1. 1). *Geoscientific Model Development* **5**, 185-191 (2012). [↑](#endnote-ref-32)
33. 33. Landrum, L., Otto-Bliesner, B. L., Wahl, E. R., Conley, A., Lawrence, P. J., Rosenbloom, N., & Teng, H. Last millennium climate and its variability in CCSM4. *J. Climate.* **26**, 1085-1111 (2013). [↑](#endnote-ref-33)
34. 34. Phipps, S. J., McGregor, H. V., Gergis, J., Gallant, A. J., Neukom, R., Stevenson, S., and Van Ommen, T. D.: Paleoclimate data–model comparison and the role of climate forcings over the past 1500 years, *J. Climate* **26**, 6915-6936 (2013). [↑](#endnote-ref-34)
35. 35. Zhou T., Li, B., Man W., Zhang, L, & Zhang, J. A Comparison of the Medieval Warm Period, Little Ice Age and 20th Century Warming simulated by the FGOALS Climate System Model. *Chin. Sci. Bull.* **56**, 3028-3041 (2011). [↑](#endnote-ref-35)
36. 36. Schmidt, G.A., Kelley, M., Nazarenko, L., Ruedy, R., Russell, G.L., Aleinov, I., Bauer, M., Bauer, S.E., Bhat, M.K., Bleck, R. & Canuto, V. Configuration and assessment of the GISS ModelE2 contributions to the CMIP5 archive. *Journal of Advances in Modeling Earth Systems* **6(1)**, 141-184 (2014). [↑](#endnote-ref-36)
37. 37. Schurer, A. P., Hegerl, G. C., Mann, M. E., Tett, S. F. B. & Phipps, S. J. Separating forced from chaotic climate variability over the Past Millennium, *J. Climate* **26**, 6954-6973 (2013). [↑](#endnote-ref-37)
38. 38. Dufresne, J.-L., Foujols, M.-A., Denvil, S., Caubel, A., Marti, O., et al. Climate change projections using the IPSL-CM5 Earth System Model: from CMIP3 to CMIP5. *Clim. Dynam.* **40**, 2123-2165 (2013). [↑](#endnote-ref-38)
39. 39. Lehner, F., Joos, F., Raible, C. C., Mignot, J., Born, A., Keller, K. M., & Stocker, T. F. Climate and carbon cycle dynamics in a CESM simulation from 850 to 2100 CE. *Earth Syst. Dynam.* **6**, 411-434 (2015). [↑](#endnote-ref-39)
40. 40. Jungclaus, J. H., Lohmann, K. & Zanchettin, D. Enhanced 20^th^ century heat transfer to the Arctic simulated in the context of climate variations over the last millennium. *Clim. Past* **10**, 2201-2213 (2014). [↑](#endnote-ref-40)
41. 41. Jungclaus, J. H., Lorenz, S. J., Timmreck, C., Reick, C. H., Brovkin, V., Six, K., Segschneider, J., Giorgetta, M. A., Crowley, T. J., Pongratz, J., Krivova, N. A., Vieira, L. E., Solanki, S. K., Klocke, D., Botzet, M., Esch, M., Gayler, V., Haak, H., Raddatz, T. J., Roeckner, E., Schnur, R., Widmann, H., Claussen, M., Stevens, B. & Marotzke, J. Climate and carbon-cycle variability over the last millennium. *Clim. Past* **6**, 723-737 (2010). [↑](#endnote-ref-41)
